# The transcription factor c-Jun/AP-1 promotes liver fibrosis during non-alcoholic steatohepatitis by regulating Osteopontin expression

**DOI:** 10.1038/s41418-018-0239-8

**Published:** 2019-02-18

**Authors:** Isabel Schulien, Birgit Hockenjos, Annette Schmitt-Graeff, Markus Große Perdekamp, Marie Follo, Robert Thimme, Peter Hasselblatt

**Affiliations:** 1 Department of Medicine II, Medical Center—University of Freiburg and Faculty of Medicine, University Hospital Freiburg, Freiburg, Germany; 2 Faculty of Biology, Albert-Ludwigs University Freiburg, Freiburg, Germany; 3 Institute of Pathology, Medical Center—University of Freiburg and Faculty of Medicine, University Hospital Freiburg, Freiburg, Germany; 4 Institute of Forensic Medicine, Medical Center—University of Freiburg and Faculty of Medicine, University Hospital Freiburg, Freiburg, Germany; 5 Department of Medicine I, Medical Center—University of Freiburg and Faculty of Medicine, University Hospital Freiburg, Freiburg, Germany

**Keywords:** Chronic inflammation, Gastrointestinal diseases, Cell biology

## Abstract

Progression of non-alcoholic fatty liver disease (NAFLD) from steatosis to non-alcoholic steatohepatitis (NASH) is a key step of NASH pathogenesis. The AP-1 transcription factor c-Jun is an important regulator of hepatic stress responses, but its contribution to NASH pathogenesis remains poorly defined. We therefore addressed c-Jun expression in liver biopsies of patients with steatosis and NASH. The role of c-Jun during NASH pathogenesis was analyzed mechanistically in *c-Jun* mutant mice fed with a methionine- and choline-deficient diet (MCDD). Disease progression from steatosis to NASH in patients correlated with increased c-Jun expression in hepatocytes, while its expression in non-parenchymal liver cells (NPLCs) particularly correlated with fibrosis. Analysis of untreated and MCDD-fed mice lacking *c-Jun* in hepatocytes (*c-Jun*^∆li^) revealed that c-Jun promotes hepatocyte survival, thereby protecting against the regenerative ductular reaction (DR) of Sox9/Osteopontin (Opn) co-expressing NPLCs, expression of the Opn receptor CD44 and fibrosis, which were all exacerbated in *c-Jun*^∆li^ mice. Since Opn and c-Jun were co-expressed by NPLCs in mice and patients with NASH, we wondered whether the increased fibrosis observed in *c-Jun*^∆li^ mice could be rescued by additional *c-Jun* deletion in NPLCs (*c-Jun*^∆li*^). *c-Jun*^∆li*^ mice with NASH indeed exhibited reduced expression of Opn and CD44 in NPLCs, impaired DR and reduced fibrosis. A similar phenotype was observed in *Opn* knockout mice, suggesting that the observed functions of c-Jun were indeed Opn-dependent. In conclusion, c-Jun expression correlates with disease progression from steatosis to NASH in patients and exerts cell-type-specific functions in mice: In hepatocytes, it promotes cell survival thereby limiting the DR and fibrogenesis. In NPLCs, it rather promotes the DR and fibrogenesis by regulating expression of Opn and CD44.

## Introduction

Non-alcoholic fatty liver disease (NAFLD) affects ~25% of the population in western countries. One quarter of patients with non-alcoholic fatty liver (NAFL) develops non-alcoholic steatohepatitis (NASH), which may further progress to liver cirrhosis and hepatocellular carcinoma (HCC) [1]. The prognosis of NASH closely correlates with the degree of liver fibrosis [2]. NASH pathogenesis is complex and involves many genetic, epigenetic and environmental factors [1]. In brief, nutritional surplus, altered intestinal microbiota and reactive oxygen species as well as free fatty acids directly cause hepatocyte damage, which in turn paves the path for compensatory mechanisms such as activation and proliferation of bipotential hepatic progenitor cells (HPC) and cholangiocytes, a phenomenon called ductular reaction (DR), inflammation involving Kupffer cells, natural killer T (NKT) cells and lymphocytes, subsequent hepatic stellate cell (HSC) activation and fibrosis [3]. However, the molecular basis of the progression from NAFL to NASH and NASH-related fibrosis is not well-understood. Moreover, the therapeutic options to treat NASH are limited. This highlights the need to better characterize the molecular steps involved in NASH pathogenesis in order to unveil novel and promising therapeutic targets.

Activator protein 1 (AP-1) is a dimeric transcription factor consisting of Jun (c-Jun, JunB and JunD), Fos (c-Fos, FosB, Fra-1 and Fra-2), activating transcription factor (Atf) and musculoaponeurotic fibrosarcoma (Maf) proteins [4]. In particular, c-Jun has been shown to play essential roles in many aspects of liver biology and disease: constitutive *c-Jun*^-/-^ knockout mice die around day E13 of embryonic development and display increased apoptosis of hepatoblasts, indicating that c-Jun expression is indispensable during liver development [5]. This phenotype could be circumvented in conditional knockout mice with hepatocyte-specific *c-Jun* deletion around birth. In these mice, it has been shown that c-Jun promotes hepatocyte proliferation as well as chemically induced and hepatitis B virus (HBV)-related hepatocarcinogenesis [6–9]. Moreover, c-Jun promotes hepatocyte survival during fulminant immune-mediated hepatitis and chemically induced endoplasmic reticulum (ER) stress [10, 11]. Several lines of evidence suggest that AP-1 and in particular c-Jun may also be involved in the pathogenesis of metabolic liver disease. It has recently been shown that overexpression of Fra-1 and Fra-2 protects against NAFL and NASH induced by a high fat diet (HFD) by suppressing transcription of *Pparγ* through the action of inhibitory c-Jun∼Fra-1 or c-Jun∼Fra-2 heterodimers [12]. In addition, several pathways involved in NASH pathogenesis, such as insulin resistance, the ER stress response and autophagy, are functionally connected by the Jun kinases Jnk1 and Jnk2 [13], which act upstream of c-Jun and determine its activity. NASH severity in MCDD-fed mice, an established mouse model of NASH and subsequent fibrosis, was profoundly reduced in *Jnk1*^-/-^ mice, which also displayed reduced hepatic c-Jun expression as well as c-Jun phosphorylation [14]. Moreover, feeding wild-type mice a NASH-inducing western diet resulted in increased c-Jun expression and profound alterations of c-Jun-dependent gene expression [15]. However, the mechanistic impact of c-Jun on NASH pathogenesis remains poorly defined. We therefore aimed to characterize its functions in NAFL, NASH and subsequent liver fibrosis in more detail. To this end and to genetically dissect the functions of c-Jun in the different liver cell compartments, mice with hepatocyte-specific knockout as well as broader *c-Jun* deletion in several tissues including both, hepatocytes and NPLCs, were generated and NASH was induced by the MCDD model, which recapitulates many hepatic features of NASH including fibrosis.

## Materials and methods

### Human liver tissue

Paraffin-embedded sections of human liver biopsies of patients previously diagnosed with NAFL, NASH and controls without liver disease were obtained from the archives of the Department of Pathology, University Hospital Freiburg, Germany. Histological scoring was performed using the NAFLD activity score (NAS; 0–8 points), fibrosis stage (0–4) and a composite score consisting of the NAS and fibrosis stage [16, 17]. Experiments involving archived patient biopsies were approved by the local ethics committee (University Hospital Freiburg, permit number 235/03).

### Animal maintenance and treatment

Mice with conditional alleles of *c-Jun* (*c-Jun*^f/f^) were crossed with transgenic *AlfpCre* mice to obtain animals with hepatocyte-specific knockout of *c-Jun* (*c-Jun*^∆li^) [6, 18]. Moreover, *c-Jun*^f/f^ mice were crossed with transgenic mice expressing Cre under the control of the interferon-responsive *Mx1* promoter. Cre-mediated recombination was induced by two intraperitoneal injections of polyinosine–polycytidylic acid (poly[I∙C], 15 µg/g body weight [BW], Amersham Bioscience, Piscataway, NJ) at least 1 week before the experiment to obtain *c-Jun*^∆li*^ mice [6, 19]. Mice were bred on a mixed genetic background (C57BL/6 × 129/Sv). C57BL/6 *Opn*^-/-^ mice were obtained from Jaxlab (www.jax.org) and maintained through heterozygous breeding. Animals were housed under specific pathogen-free conditions at 22 °C under controlled 12 h light/dark cycle and had access to autoclaved water ad libitum. Only male mice were used throughout the studies, and mice were randomized into the treatment groups. Littermate controls not expressing Cre (*c-Jun*^f/f^) or *Tg(AlfpCre) c-Jun*^*+/+*^ were used for *c-Jun* mutants, while *Opn*^+/+^ and *Opn*^+/-^ mice were used as controls for *Opn*^-/-^ mice. At 6 weeks of age, mice were placed on a high fat diet (ssniff^®^ Spezialdiäten GmbH, Soest, Germany) for 84 days to induce NAFL, on a MCDD (ssniff^®^) for 14 and 49 days to induce NASH or on a control diet (CD). All animals received human care and experiments were performed in accordance with local and institutional regulations and approved by the local animal ethics committee (Regierungspräsidium Freiburg, Germany, permit number G-15/091) and reported according to the ARRIVE guideline. Exact sample size for each experimental group is indicated in the Figure legends.

### Cell culture

Isolation of primary murine hepatocytes (PMH) was performed as previously described [11]. PMH were infected by adenovirus carrying the Cre recombinase gene and GFP or only GFP (Gene Transfer Vector Core Facility, University of Iowa, Iowa, IA, USA; 10 MOI) for 3 h and collected after 24 h.

### Serum analyses

Serum alanine transaminase (ALT) activities were measured using clinical routine assays at the University Hospital Freiburg.

### Histology, immunohistochemistry and -fluorescence

For histology, livers were fixed in 3.7% neutral buffered formaldehyde at 4 °C and embedded in paraffin. Paraffin-embedded liver tissues were stained with H&E for morphological analysis. Immunohistochemistry was performed using the Envision kit (Dako, Hamburg, Germany) and/or Warp Red Chromogen kit (Biocare Medical, Pacheco, CA, USA). For c-Jun, cleaved-caspase 3, Sox9, Ki67, NKp46, CD3, F4/80 and Ly6G quantification, the number of positively stained cells in 15 randomly selected high-power fields was determined. Paraffin-embedded sections were stained with Sirius Red staining solution and the Sirius Red-positive area of 15 randomly selected 200x high-power fields was analyzed via colour error measurement using Image-J software (National Institute of Health, Bethesda, MD, USA). TUNEL (terminal deoxynucleotidyl transferase dUTP nick-end labelling) assay (Roche, Indianapolis, IN, USA), and immunofluorescence were performed on paraffin-embedded liver tissues and DAPI was used to visualize cell nuclei. Three liver lobes per mouse were scanned in their entirety on an Olympus ScanR IX81 inverted microscope using a 20 × 0.45 LUCPLFLN objective. The percentage of TUNEL-positive nuclei was determined using the Olympus ScanR analysis software. For Oil-Red-O stainings frozen liver tissues were stained with staining Solution (Sigma). A detailed list of the antibodies used can be found in the Supplementary Information (Supplementary Table 1).

### RNA and qPCR

For isolation of total RNA, PMHs or liver tissues snap frozen in liquid nitrogen were lysed using QIAshredder and the RNeasy^®^ Mini Kit (Qiagen, Hilden, Germany). Complementary DNA synthesis was performed using the First Strand cDNA synthesis kit (Fermentas, St. Leon-Rot, Germany). qPCR was performed with SYBR Green (Invitrogen, Karlsruhe, Germany) and 10% dimethylsulphoxide (Sigma) on a Lightcycler 480 (Roche; 40 cycles: 30” 95 °C; 30” 60 °C; 40” 72 °C). Loading was normalized to 18S ribosomal RNA and actin messenger RNA. Expression was normalized to untreated controls. All primers were designed using Primer-BLAST (www.ncbi.nlm.nih.gov/tools/primer-blast). Primers were synthesized by Microsynth, Balgach, Switzerland, and specificity of the PCR products was analyzed by melting curve analysis. Primer sequences are listed in the Supplementary Information (Supplementary Table 2).

### Western blot analysis

Total liver lysates were analyzed by immunoblot using antibodies for c-Jun and β-actin. A detailed list of the antibodies used can be found in the Supplementary Information (Supplementary Tabel 1).

### Statistics

Data in bar graphs represent mean ± SD, data in dot plots are depicted with their mean and data in boxplots with median ± min/max. Normal distribution of the data was tested using GraphPad PRISM software (La Jolla, CA, USA). Statistical analysis was performed using the non-parametric Mann–Whitney test for the comparison of MCDD-treated mice with different genotypes at a pre-specified timepoint. However, in some experiments, analysis of untreated mice was included in an explorative manner, and it should be noted that the sample size of these untreated animals was not large enough to enable multiple comparisons with corrections for multiple testing. Correlation analysis was performed using the Pearson correlation coefficient. Statistical analyses were performed using GraphPad PRISM.

## Results

### Expression of c-Jun increases with disease progression from steatosis to NASH

**Fig. 1:**
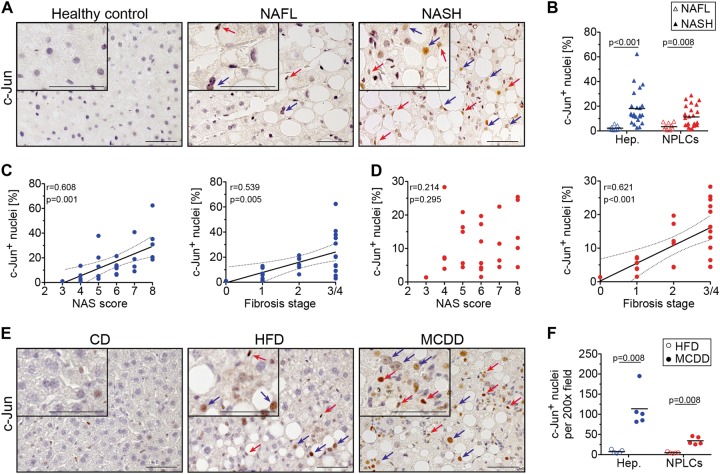
c-Jun expression correlates with disease progression from NAFL to NASH in humans and mice. **a** Representative immunohistochemistry for c-Jun of human liver biopsies from healthy controls, patients with NAFL or NASH. **b** c-Jun expression in hepatocytes and NPLCs of NAFL (*n* = 9) and NASH patients (*n* = 27) was quantified. **c** Hepatocellular c-Jun expression was correlated with the NAS (**c**, left panel) and fibrosis stage (**c**, right panel). **d** c-Jun expression in NPLCs was correlated with the NAS (**d**, left panel) and fibrosis stage (**d**, right panel). **e** Representative immunohistochemistry for c-Jun of livers from mice treated with a CD, HFD or MCDD. **f** c-Jun expression in hepatocytes and NPLCs of HFD- (*n* = 4) and MCDD-fed mice (*n* = 5) was quantified. Significance was tested by Mann–Whitney test and correlation was tested by Pearson correlation coefficient. p values are indicated if significant. Scale bar = 50 µm. Hepatocytes and NPLCs are depicted by blue and red arrows, respectively

In order to analyse the expression of c-Jun at various stages of metabolic liver disease, immunohistochemistry was performed on liver sections from individuals without liver disease (*n* = 5) and patients with NAFL (i.e., steatosis) (*n* = 9) or NASH (*n* = 27). Disease severity was determined histologically using the NAFLD activity score (NAS, 0–8 points, addressing steatosis, hepatocyte ballooning and lobular inflammation) [16], fibrosis stage (0–4) and a composite NASH score (sum of NAS plus fibrosis stage) [17]. While c-Jun expression was absent in healthy livers as previously shown [10], patients with NAFL exhibited moderate nuclear c-Jun expression in hepatocytes and NPLCs, which was substantially increased in patients with NASH (Fig. 1a, b). Moreover, hepatocellular c-Jun expression correlated significantly with the NAS, fibrosis stage and composite NASH score (Fig. 1c, Supplementary Fig. 1A). In contrast, c-Jun expression in NPLCs only correlated with fibrosis stage, but not with the NAS or composite NASH score (Fig. 1d, Supplementary Fig. 1B), suggesting that c-Jun expression in NPLCs may be particularly involved in fibrogenesis. Feeding of wild-type mice with control, high fat or MCD diet resulted in similar expression patterns with c-Jun expression being most strongly induced in hepatocytes and NPLCs during MCDD-related NASH and, to a lesser extent, during HFD-related steatosis (Fig. 1e, f). Therefore, the MCDD mouse model was chosen to further unravel the functions of c-Jun during NASH pathogenesis, since it recapitulates many hepatic features of NASH including fibrosis.

### Increased fibrosis in MCDD-treated mice lacking *c-Jun* expression in hepatocytes

**Fig. 2:**
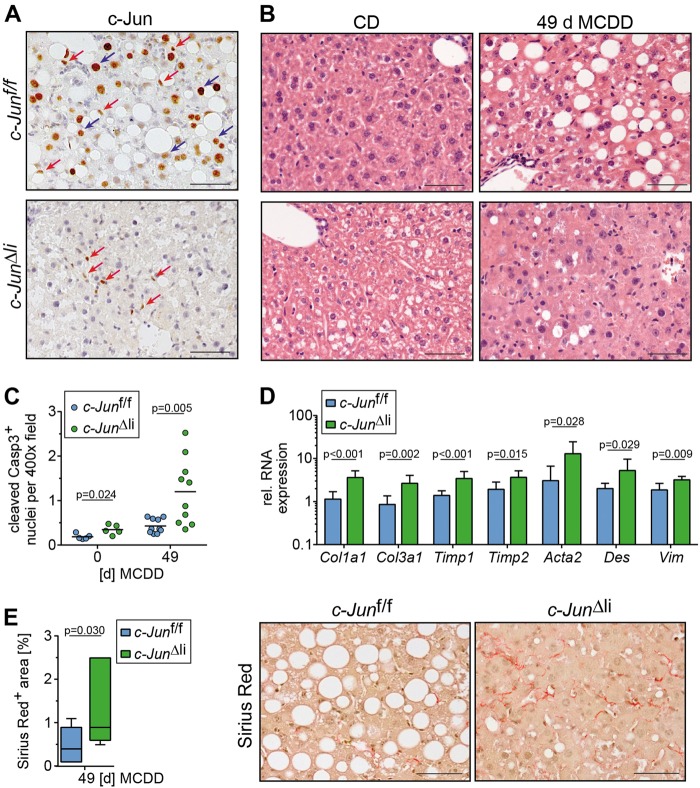
Increased fibrosis in MCDD-fed *c-Jun*^∆li^ mice. **a, b** Representative immunohistochemistry for c-Jun following 49 days of MCDD feeding (**a**) and hematoxylin and eosin (H&E) stainings following 0 and 49 days of MCDD feeding (**b**) of livers from mice with the indicated genotypes. Nuclei of hepatocytes and NPLCs are depicted by blue and red arrows, respectively. **c** Cells with caspase 3 cleavage were assessed by immunofluorescence and quantified (n = 5-10/genotype and timepoint). **d** Hepatic mRNA expression of the indicated genes following 49 days of MCDD (*n* = 5-8/genotype). mRNA expression is shown relative to untreated controls. **e** Quantification and representative Sirius Red stainings following 49 days of MCDD (left, *n* = 7/genotype). Significance was tested by Mann–Whitney test. *P*-values are indicated if significant. Scale bar = 50 µm

**Fig. 3:**
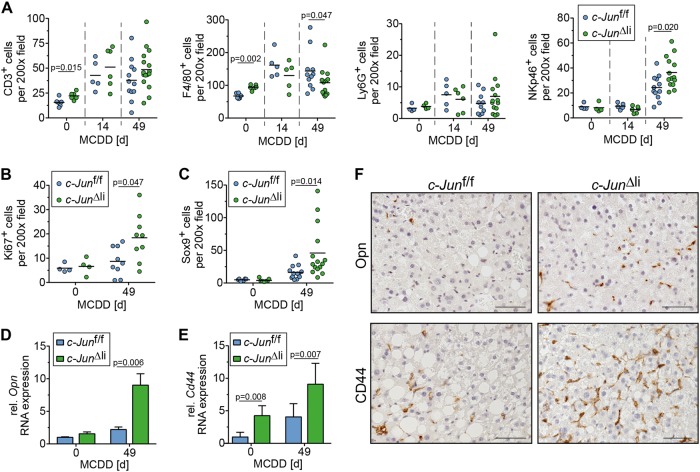
Increased DR in MCDD-fed *c-Jun*^∆li^ mice. **a** The number of CD3-, F4/80-, Ly6G-, and NKp46-positive cells was assessed by immunohistochemistry (*n* = 4-16/genotype and timepoint). **b** Quantification of Ki67-positive NPLCs by immunohistochemistry (*n* = 4–9/genotype and timepoint). **c** Sox9-positive cells were assessed by immunohistochemistry (*n* = 5–15/genotype and timepoint). **d**–**f** Hepatic expression of Opn and of its receptor CD44 was determined by qPCR (**d** and **e**, *n* = 4–8/genotype and timepoint) and immunohistochemistry (**f**, representative stainings following 49 days of MCDD). mRNA expression is shown relative to untreated controls. Significance was tested by Mann–Whitney test. *P*-values are indicated if significant. Scale bar = 50 µm

MCDD feeding of *c-Jun*^f/f^ control mice resulted in strongly induced nuclear c-Jun expression in both, hepatocytes as well as NPLCs (Fig. 2a). c-Jun expression was absent in hepatocytes of *c-Jun*^Δli^ mice as determined by immunohistochemistry and immunoblotting of total liver lysates, thus confirming a high recombination efficiency (Fig. 2a, Supplementary Fig. 1C). mRNA expression of *c-Fos*, *Fra-1* and *Fra-2*, which have been reported to form heterodimers with c-Jun during NASH [12], was not affected in *c-Jun*^Δli^ as compared with *c-Jun*^f/f^ livers (Supplementary Fig. 2A). The expression of genes related to lipid metabolism was not affected by the loss of *c-Jun* during early stages of MCDD-mediated NASH (Supplementary Fig. 2B). Histological analysis of *c-Jun*^Δli^ mice treated with MCDD for 49 days revealed reduced steatosis (Supplementary Fig. 2C) and more heterogeneous hepatocyte morphology (Fig. 2b). Interestingly, reduced steatosis did not correlate with an amelioration of NASH, but was rather a consequence of increased fibrosis (see below), as previously described in NASH patients [20]. Moreover, hepatocellular apoptosis was increased in MCDD-treated *c-Jun*^Δli^ mice as determined by staining for cleaved-caspase 3 (Fig. 2c, Supplementary Fig. 3A), although the absolute numbers of apoptotic cells were low and did not cause differences in serum alanine aminotransferase (ALT) concentrations as compared with MCDD-treated controls (Supplementary Fig. 3B). However, MCDD feeding of *c-Jun*^∆li^ mice also resulted in increased expression of fibrogenesis-related genes (Fig. 2d) and subsequently increased fibrosis as determined by Sirius red staining (Fig. 2e). Interestingly, caspase cleavage was already slightly increased in untreated *c-Jun*^Δli^ mice (Fig. 2c), in which serum ALT concentrations (Supplementary Fig. 3B) as well as inflammation as evidenced by increased numbers of CD3^+^ T cells and F4/80^+^ macrophages were slightly increased (Fig. 3a). These alterations also correlated with increased expression of fibrogenesis-related genes including *Col1a1* and *Tgfb1* in untreated *c-Jun*^∆li^ mice (Supplementary Fig. 3C).

c-Jun is an important regulator of hepatocyte survival in chemically induced ER stress and there are several indications that NAFLD-mediated lipotoxicity and deregulated ER stress responses are closely connected [11, 21–23]. We therefore analysed the expression of ER stress markers in MCDD-fed mice. Although hepatic expression of *Gadd153* mRNA and *Hspa5* protein (also known as *Grp78* or *BiP*), was induced in livers of MCDD-fed mice, their expression was not altered in the absence of hepatocellular *c-Jun* (Supplementary Fig. 3D). To further analyse potential functions of c-Jun during lipotoxicity in vitro, *c-Jun*^f/f^ PMHs were isolated and recombination of *c-Jun* was mediated by infection with adenoviral vectors expressing Cre recombinase. Incubation of Adeno-GFP-infected control PMHs with palmitate resulted in lipotoxicity, concentration-dependent increase of TUNEL-positive apoptotic cells, as well as release of ALT into the supernatant. However, these alterations were not affected by additional recombination of *c-Jun* (Supplementary Fig. 4A, B). These findings suggest that c-Jun is not involved in regulating the hepatic ER stress response in MCDD-fed mice and has only limited impact on regulating hepatocyte survival, at least during the more prominent degrees of lipotoxicity studied here in vitro and in the MCDD model.

Since insertion of the *AlfpCre* transgene was previously shown to affect the expression patterns of genes involved in lipid metabolism [24], untreated and MCDD-treated *Tg(AlfpCre) c-Jun*^+/+^ and *c-Jun*^+/+^ mice were analysed as additional controls. ALT concentrations, hepatic caspase 3 cleavage, liver inflammation, fibrosis and expression of fibrogenesis-related genes were comparable in *Tg(AlfpCre) c-Jun*^+/+^ and *c-Jun*^+/+^ mice in each, untreated or MCDD-treated conditions (Supplementary Fig. 5A–D). These findings suggest that the moderate hepatitis observed in untreated *c-Jun*^Δli^ mice occurs independent of the AlfpCre transgene and results in moderate liver injury which may serve as a “first hit” and sensitize the liver to the more pronounced MCDD-mediated fibrosis.

### Increased fibrosis in *c-Jun*^∆li^ mice correlates with an increased ductular reaction

A regenerative response called ductular reaction (DR) involving cholangiocytes, HPCs and inflammatory cells, precedes liver fibrosis [2]. Indeed, proliferation of NPLCs was increased in MCDD-treated *c-Jun*^Δli^ mice (Fig. 3b), consistent with increased DR. Moreover, NPLC expression of Sox9, a marker for HPCs and cholangiocytes [25], was significantly increased as compared with MCDD-fed *c-Jun*^f/f^ littermates and *Tg(AlfpCre)c-Jun*^+/+^ controls (Fig. 3c, Supplementary Fig. 5E). The pro-inflammatory cytokine Osteopontin (Opn, also known as Spp1) is another established marker of the DR in NASH patients and MCDD-fed mice [26–28]. Analysis of hepatic mRNA expression and immunohistochemistry revealed that Opn expression was strongly induced in *c-Jun*^∆li^ mice at later stages of the MCDD protocol and that its expression was predominantly localized in NPLCs (Fig. 3d, f, Supplementary Fig. 5E). In keeping with this notion, expression of the Opn receptor CD44 was also induced in livers of *c-Jun*^∆li^ mice (Fig. 3e, f, Supplementary Fig. 5E). Since Opn and CD44 exert pro-inflammatory effects during NASH pathogenesis [29, 30], infiltration of inflammatory cells was examined by immunohistochemistry. Numbers of hepatic Ly6G^+^ neutrophils and CD3^+^ T cells were increased upon MCDD feeding, but not affected by the deletion of *c-Jun* (Fig. 3a). In contrast, numbers of hepatic F4/80^+^ macrophages and monocytes were slightly reduced in MCDD-fed *c-Jun*^∆li^ mice at late stages of MCDD feeding (Fig. 3a) while numbers of hepatic NKp46^+^ natural killer (NK) and NKT cells, a major cellular source of Opn expression [31], were increased in *c-Jun*^∆li^ mice during late stages of the MCDD protocol (Fig. 3a).

**Fig. 4:**
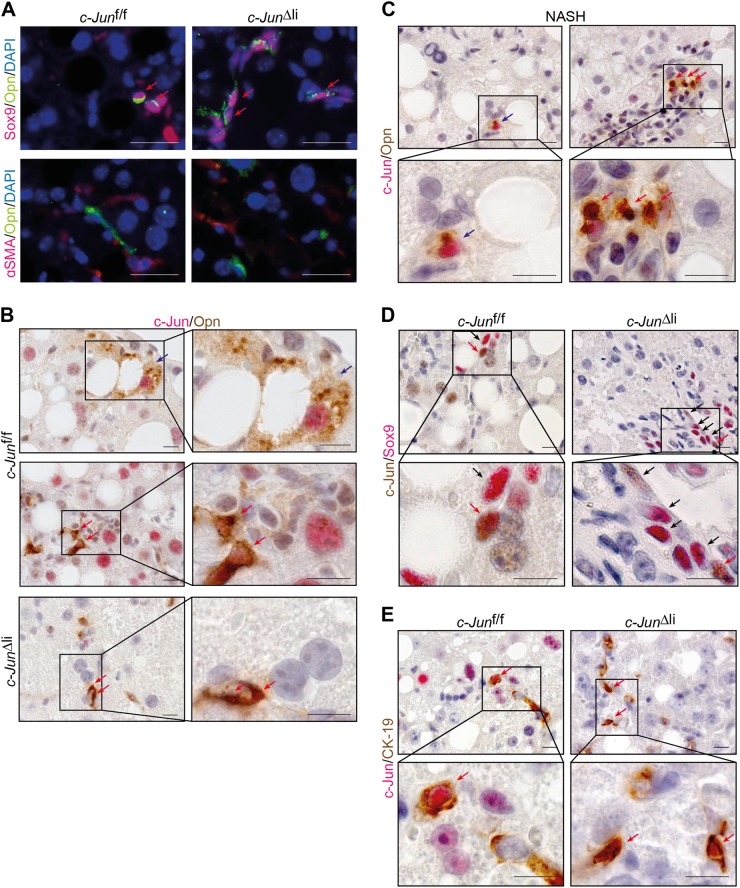
Co-expression of c-Jun with the HPC markers Sox9, CK-19 and Opn. **a** Representative double-immunofluorescence stainings for Opn (green) and Sox9 (red) (upper panel) or αSMA (red) (lower panel) of livers from mice with the indicated genotypes following 49 days of MCDD feeding. Double-positive cells are indicated by red arrows. **b, c** Representative immunohistochemistry of OPN (brown) and c-Jun (red) double-positive cells of mice of the indicated genotypes following 49 days of MCDD feeding (**b**) or liver sections of patients with NASH (**c**). **d, e** Representative immunohistochemistry of c-Jun (**d** = brown, **e** = red) and Sox9 (**d** = red) or CK-19 (**e** = brown) double-positive cells of mice of the indicated genotypes following 49 days of MCDD feeding. Double-positive hepatocytes and NPLCs are indicated by blue and red arrows, respectively. Single positive cells are indicated by black arrows. Scale bar (immunofluorescence) = 25 µm. Scale bar (immunohistochemistry) = 10 µm

**Fig. 5:**
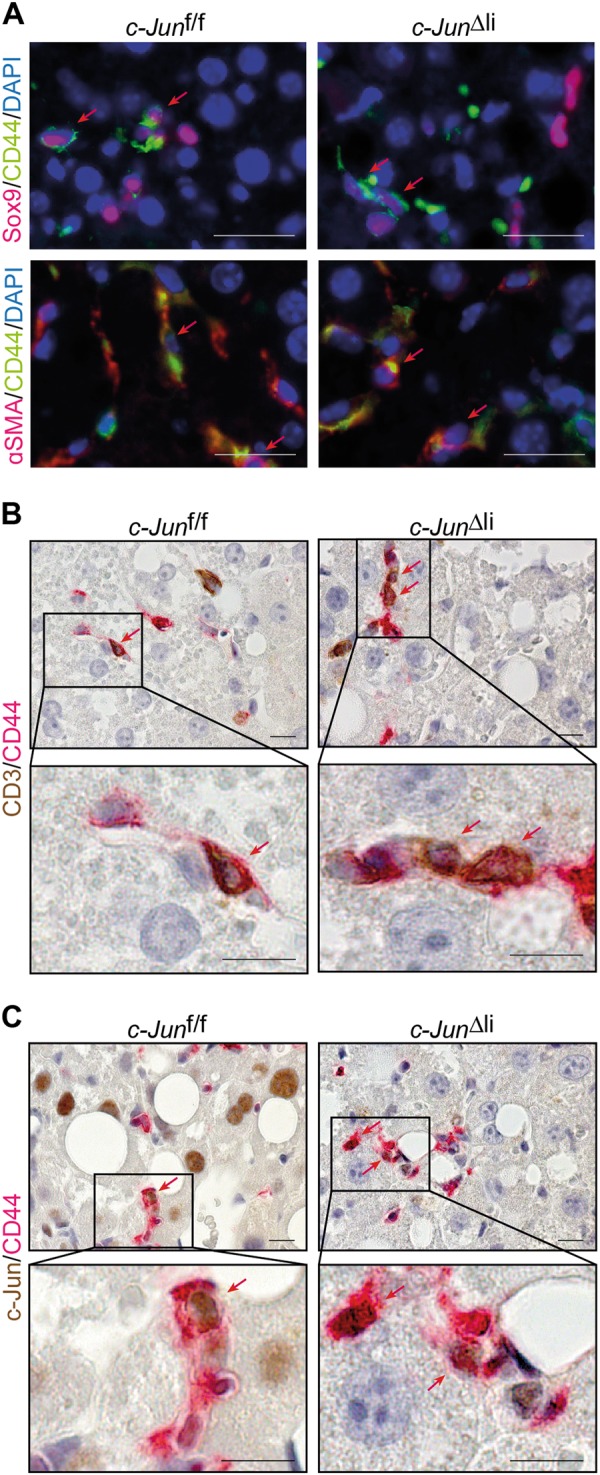
Expression analysis of CD44-positive NPLCs. **a** Representative double-immunofluorescence stainings for CD44 (green) and Sox9 (red) (upper panel) or αSMA (red) (lower panel) of livers from mice with the indicated genotypes following 49 days of MCDD feeding. **b, c** Representative immunohistochemistry of CD44 (red) and CD3 (**b** = brown) or c-Jun (**c** **=** brown) double-positive cells of livers from mice with the indicated genotypes following 49 days of MCDD feeding. Double-positive cells are indicated by red arrows. Scale bar (immunofluorescence) = 25 µm. Scale bar (immunohistochemistry) = 10 µm

It was previously shown in HCC cancer stem cells that Opn may be controlled by Sox9 [32]. However, Sox9 and Opn may also be expressed by hepatic stellate cells (HSC) while increased Opn expression in Sox9-negative cells could be mediated by NKT cells [33, 34]. Opn expressing NPLCs were therefore analysed in more detail by double-immunofluorescence stainings, which revealed that Opn was closely co-expressed with Sox9 (Fig. 4a, Supplementary Fig. 6A), but not with the HSC marker α smooth muscle actin (αSMA) (Fig. 4a, Supplementary Fig. 6B), suggesting that increased Opn expression in *c-Jun*^∆li^ livers was most likely mediated by HPCs. *Opn* and *CD44* are both established AP-1 target genes. Expression of both genes was consistently reduced in PMH following Adeno-Cre-mediated recombination of *c-Jun* (Supplementary Fig. 4C), suggesting that c-Jun regulates the expression of these genes in a cell-autonomous manner, at least in hepatocytes. Moreover, co-expression of c-Jun and Opn was evident in hepatocytes and NPLCs of MCDD-fed control mice and NPLCs of *c-Jun*^∆li^ mice (Fig. 4b) as well as in hepatocytes and NPLCs of patients with NASH (Fig. 4c). The majority of Sox9-positive cells co-expressed cytokeratin 19 (CK-19), another marker for cholangiocytes and HPCs (Supplementary Fig. 6C). Immunohistochemistry revealed co-localization of c-Jun with both Sox9 (Fig. 4d) and CK-19 (Fig. 4e) in livers of MCDD-fed control and *c-Jun*^∆li^ mice. This co-expression of Sox9, CK-19, Opn and c-Jun during the DR is consistent with expression of these proteins in HPCs. Analysis of CD44 expression revealed that CD44 was co-expressed with Sox9 (Fig. 5a, Supplementary Fig. 7A), αSMA (Fig. 5a, Supplementary Fig. 7B), CD3 (Fig. 5b), as well as with c-Jun (Fig. 5c), suggesting that increased CD44 expression occurred in HPCs, activated stellate cells and lymphocytes and may be associated with c-Jun expression. These findings suggest that c-Jun expression in NPLCs promotes the DR by regulating the expression of Opn in Sox9-positive HPCs and of CD44 in HPCs, stellate cells and lymphocytes.

### Increased Opn expression, DR and fibrosis are rescued in *c-Jun*^∆li*^ mice

**Fig. 6:**
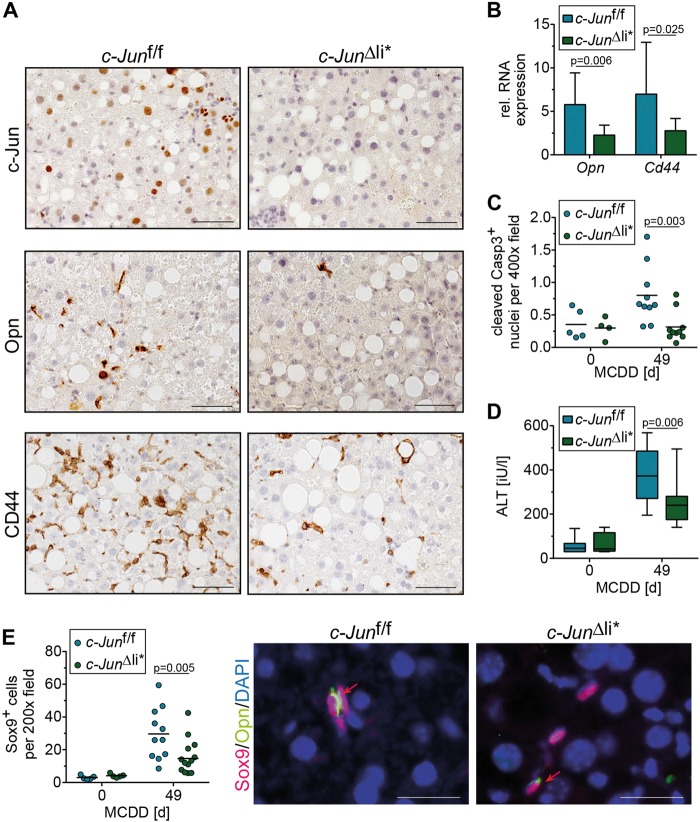
c-Jun promotes Opn expression in NPLCs and the subsequent DR. **a** Representative immunohistochemical stainings for c-Jun, OPN and CD44 of liver sections from mice with the indicated genotype following 49 days of MCDD. **b** Hepatic *Opn* and *Cd44* mRNA expression (*n* = 5–13/genotype and timepoint). mRNA expression is shown relative to untreated controls. **c** Caspase 3 cleavage was assessed by immunofluorescence (n = 4-10/genotype and timepoint). **d** Serum ALT concentrations (*n* = 4–12/genotype and timepoint). **e** Quantification of Sox9-positive cells by immunohistochemistry (left, *n* = 5–13/genotype and timepoint). Representative double-immunofluorescence stainings for Sox9 (red) and Opn (green) of livers from mice with the indicated genotype following 49 days of MCDD feeding are shown (right). Double-positive cells are indicated by red arrows. Significance was tested by Mann–Whitney test. *P*-values are indicated if significant. Scale bar (immunofluorescence) = 25 µm. Scale bar (immunohistochemistry) = 50 µm

**Fig. 7:**
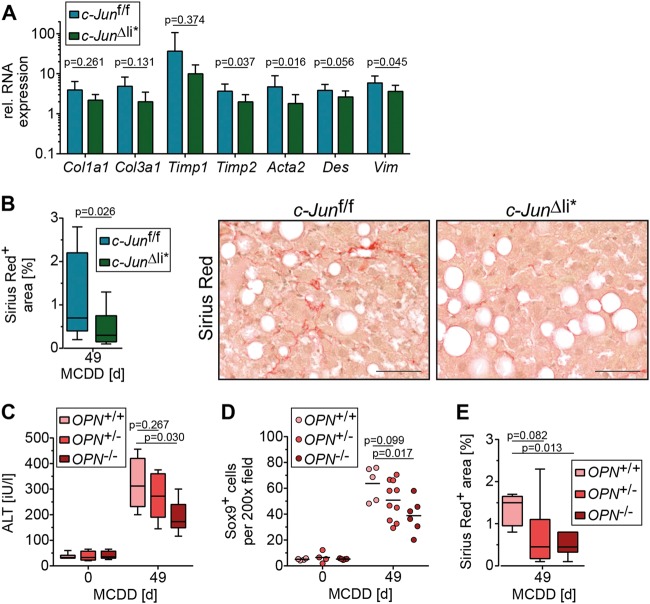
c-Jun expression in NPLCs promotes fibrosis by regulating Opn expression. **a** Hepatic mRNA expression of the depicted genes following 49 days of MCDD (*n* = 5–13/genotype). mRNA expression is shown relative to untreated controls. **b** Quantification of Sirius red-stained area following 49 days of MCDD (left panel; *n* = 11–12/genotype) and representative stainings (right panel). **c** Serum ALT concentrations of mice with the indicated genotypes treated with CD or MCDD (*n* = 4–8/genotype and timepoint). **d** Quantification of Sox9-positive cells by immunohistochemistry (*n* = 4–10/genotype and timepoint). **e** Quantification of the Sirius red-positive area following 49 days of MCDD (*n* = 5–6/genotype). Significance was tested by Mann–Whitney test. *P*-values are indicated. Scale bar = 50 µm

Our findings raise the question, as to whether the increased DR, Opn expression and fibrosis could be rescued by knockout of *c-Jun* in NPLCs. To this end, mice with broader deletion of *c-Jun*, including hepatocytes and NPLCs (*c-Jun*^∆li*^), were treated with MCDD. While loss of *c-Jun* around birth in untreated *c-Jun*^∆li^ mice was previously shown to moderately affect liver development, *c-Jun*^∆li*^ mice with *c-Jun* deletion after 6 weeks of age were phenotypically normal under resting conditions [6]. Immunohistochemistry confirmed the absence of *c-Jun* expression in both hepatic cell compartments of MCDD-fed *c-Jun*^∆li*^ mice (Fig. 6a). The amount of steatosis, hepatocyte ballooning, and inflammation was similar in MCDD-fed *c-Jun*^∆li*^ mice and *c-Jun*^f/f^ controls (Supplementary Fig. 8A, B). Expression of Opn and CD44 was strongly reduced in *c-Jun*^∆li*^ livers (Fig. 6a, b). Moreover, liver damage as determined by caspase 3 cleavage and serum ALT concentrations was reduced in *c-Jun*^∆li*^ mice (Fig. 6c, d). Reduced liver damage also correlated with impaired proliferation of NPLCs (Supplementary Fig. 8C), numbers of Sox9 expressing cells (Fig. 6e), as well as the absence of Sox9/Opn (Fig. 6e, Supplementary Fig. 9A) and αSMA/Opn co-expressing cells (Supplementary Fig. 9B). In keeping with this notion, c-Jun/Sox9 (Supplementary Fig. 10A), c-Jun/CK-19 (Supplementary Fig. 10B) and c-Jun/CD44 (Supplementary Fig. 10C) co-expressing cells were not detectable in MCDD-treated *c-Jun*^∆li*^ livers. Furthermore, the decreased expression of CD44 in MCDD-treated *c-Jun*^∆li*^ livers also correlated with reduced numbers of CD44/Sox9 (Supplementary Fig. 11A) and CD44/αSMA co-expressing cells (Supplementary Fig. 11B). Moreover, expression of fibrogenesis-related genes was reduced in *c-Jun*^∆li*^ livers, which correlated with reduced fibrosis (Fig. 7a, b). To address whether these alterations were indeed Opn-dependent, NASH was also induced in *Opn*^-/-^ mice. While MCDD treatment resulted in a comparable amount of steatosis in *Opn*^-/-^ mice as compared with the *Opn*^+/+^ and *Opn*^+/-^ controls (Supplementary Fig. 12A), liver damage as determined by serum ALT concentrations was reduced in *Opn*^-/-^ mice to a similar extent as in *c-Jun*^∆li*^ mice (Fig. 7c). Moreover, the DR of Sox9^+^ cells, liver fibrosis and infiltration of NKp46^+^ cells was significantly reduced in the absence of *Opn* (Fig. 7d, e; Supplementary Fig. 12B). These findings strongly suggest that c-Jun expression in NPLCs promotes NASH-related DR and subsequent fibrosis by regulating the expression of Opn.

## Discussion

In this study, we demonstrate that expression of the AP-1 transcription factor c-Jun correlates with progression from steatosis to NASH in humans and mice. This is consistent with previous observations of increased c-Jun expression in patients and mouse models of metabolic liver disease [14, 15, 35]. However, the functional relevance of c-Jun during NASH pathogenesis remained elusive to date. Here, we applied the MCDD protocol to three mouse strains to dissect the functions of c-Jun within distinct liver cell compartments. The transgenic Cre lines used here have been particularly useful to dissect the antagonistic and cell-type-specific functions of EGFR during liver carcinogenesis [36]. Although the MCDD model does not exhibit all hallmarks of the metabolic syndrome and NASH, such as overweight and impaired peripheral insulin sensitivity, it represents a fast and reproducible means to induce steatosis, hepatocyte ballooning and fibrosis and has therefore been extensively used to study NASH [37].

We demonstrate that untreated mice lacking *c-Jun* in hepatocytes develop mild hepatitis, which may serve as first hit in the series of events predisposing to NASH. These findings are consistent with previous reports demonstrating that c-Jun promotes hepatocyte survival in other stress conditions [7, 10, 11]. However, in the light of these findings, it was unexpected that loss of *c-Jun* did not further exacerbate cell death under conditions of increased lipotoxicity in vitro and in vivo. Despite of the mild differences in cell death, the amount of the DR was increased in MCDD-fed *c-Jun*^∆li^, but reduced in *c-Jun*^∆li*^ mice. These findings suggest that the NASH-related protective functions of c-Jun within hepatocytes are rather limited. The DR is an essential driver of fibrogenesis [2]. Our data indicate that c-Jun expression in NPLCs promotes the DR and fibrogenesis, which is most likely mediated by regulation of Opn expression. Expression of Opn and its receptor CD44 correlates with disease activity and progression of NASH in patients and mice [38] and regulates the DR and fibrosis [28, 29]. Inhibition of Opn was shown to impair regenerative HPC responses and to abrogate fibrosis [26]. The latter effect may be mediated by directly interacting with the high-mobility group box-1 axis in HSCs [39]. *Opn* is an established AP-1 target gene [9, 40, 41]. Its expression was consistently downregulated in hepatocytes following Adeno-Cre-mediated recombination of *c-Jun*, suggesting a direct and cell-autonomous interaction of c-Jun with *Opn* transcription. Moreover, co-expression of c-Jun and Opn was observed in both hepatocytes and NPLCs. Importantly, expression of Opn and CD44, as well as the fibrosis observed in *c-Jun*^∆li^ mice was reversed by additional deletion of *c-Jun* in NPLCs. It has been shown that Opn expression during NASH and subsequent fibrosis is also regulated by Hedgehog signalling [27, 42]. Expression of Hedgehog and AP-1 share many common triggers and are consecutively expressed during acute and chronic liver disease, suggesting that both pathways may indeed be involved in regulating the hepatic response to a putative “second hit” of liver damage required for the pathogenesis of NASH [1]. The functions of c-Jun in NPLCs have not been studied to date. Our finding that c-Jun rather promotes NASH and fibrosis somehow antagonizing its effects in hepatocytes adds a novel level of complexity to AP-1-related signalling pathways in the liver.

The relevance of the c-Jun/Opn axis is further highlighted by the observation that MCDD-fed *Opn*^-/-^ and *c-Jun*^∆li*^ mice displayed similar hepatoprotective phenotypes. Moreover, Opn expression correlates with disease progression in NASH patients [38] and co-localized with c-Jun in hepatocytes and NPLCs of NASH patients, strongly suggesting that the genetic links observed here in mice also apply to human disease. Recent findings identified the Opn receptor CD44 as another key regulator of NASH, in particular by promoting the transition from NAFL to NASH [30]. Our observation that CD44 expression was c-Jun-dependent adds AP-1 as a central regulator to this signalling network, which is consistent with previous findings during hepatocarcinogenesis [43].

In conclusion, c-Jun expression correlates with disease progression from steatosis to NASH in patients and exerts cell-type-specific functions in mice: In hepatocytes, it promotes cell survival thereby limiting the DR and fibrogenesis. In NPLCs, it rather promotes the DR and fibrogenesis by regulating expression of Opn and CD44. Targeting of c-Jun and Opn specifically in NPLCs may therefore be a promising therapeutic approach for NASH and its complications.

## Electronic supplementary material

Supplementary figure legends

Supplementary Tables

suppl. Fig.1

suppl. Fig.2

suppl. Fig.3

suppl. Fig.4

suppl. Fig.5

suppl. Fig.6

suppl. Fig.7

suppl. Fig.8

suppl. Fig.9

suppl. Fig.10

suppl. Fig.11

suppl. Fig.12
