# Supplementary material for: The transcription factor c-Jun/AP-1 promotes liver fibrosis during non-alcoholic steatohepatitis by regulating Osteopontin expression
Source: Cell Death Differ. 2019 Feb 18;26(9):1688–99. doi: 10.1038/s41418-018-0239-8 (PMC6748141; doi:10.1038/s41418-018-0239-8)
Supplement: Supplementary file 1 — Supplementary figure legends [file 41418_2018_239_MOESM1_ESM.docx]

**Supplementary Figure Legends**

**Supplementary Fig.1. c-Jun expression in human patients with NAFL and NASH**

(**A, B**) c-Jun expression in hepatocytes (**A**) and NPLCs (**B**) was correlated with the composite NASH score. Significance was tested using Pearson correlation coefficient analysis. p values are indicated. (**C**) c-Jun expression of total liver lysates was analyzed by western blot in mice with the indicated genotype following 49 days of MCDD. Actin was used as loading control.

**Supplementary Fig.2. Analysis of hepatic lipid accumulation in MCDD-fed *c-Jun*^Δli^ mice**

(**A**) Hepatic mRNA expression of the indicated genes was analyzed by qPCR (n=5-8/genotype and timepoint). (**B**) Hepatic mRNA expression of genes related to lipid metabolism of livers from mice with the indicated genotypes following 49 days of MCDD feeding was analyzed by qPCR (n=5/genotype). RNA expression is shown relative to untreated controls. (**C**) Representative Oil-Red-O stainings of livers from mice with the indicated genotypes and timepoints. Scale bar=20 µm.

**Supplementary Fig.3. Characterization of MCDD-fed *c-Jun*^Δli^ mice**

(**A**) Representative immunofluorescence stainings for cleaved caspase 3 of livers from mice with the indicated genotypes following 49 days of MCDD feeding. Positive cells are indicated by white arrows. (**B**) Liver damage was determined by serum ALT concentrations (n=5-16/genotype and timepoint). (**C**) Hepatic mRNA expression of the depicted genes was analyzed in mice on control diet (n=5/genotype). (**D**) Hepatic mRNA expression of the ER stress-related gene *Gadd153* and protein expression of Hspa5 were determined by qPCR (n=5-8/genotype and timepoint) and immunohistochemistry, respectively. Hspa5 positive cells are indicated by black arrows. RNA expression is shown relative to untreated controls. Significance was tested by Mann-Whitney test. p values are indicated if significant. Scale bar (immunofluorescence)=25 µm. Scale bar (immunohistochemistry)=20 µm.

**Supplementary Fig.4. c-Jun is not essential during palmitate-induced lipotoxicity *in vitro*, but regulates expression of *Opn* and *Cd44* in a cell-autonomous manner**

(**A,B**) *c-Jun*^f/f^ PMHs were infected with Adeno-GPF or –Cre and treated with 400 or 800 µM palmitate (PA) to induce lipotoxicity (n=3 per treatment). (**A**) TUNEL assays for apoptotic cells were performed and the percentage of TUNEL-positive cells quantified. (**B**) Cell damage was determined by analysis of ALT concentrations in the supernatant (results were normalized to total cell number). (**C**) *c-Jun*, *Opn* and *CD44* RNA expression was determined by qPCR in *c-Jun*^f/f^ PMHs following adenoviral transfer of Cre recombinase or GFP (n=3-5/treatment; expression was normalized to that observed in control PMHs). RNA expression is shown relative to AdenoGFP treated controls. Significance was tested by Mann-Whitney test. p values are indicated if significant.

**Supplementary Fig.5. Phenotype of MCDD-fed *Tg(AlfpCre) c-Jun*^+/+^ controls.**

(**A**) ALT concentrations (left panel, n=4-8/genotype and timepoint). Cells with cleaved caspase 3 were assessed by immunofluorescence (right panel, n=4/genotype and timepoint). (**B**) The number of the indicated immune cells was analyzed by immunohistochemistry and quantified (n=3-6/genotype and timepoint). (**C**) Hepatic mRNA expression of the depicted genes was analyzed in mice on control diet (upper panel, n=5/genotype) or following 49 days of MCDD (lower panel, n=5-6/genotype and timepoint). (**D**) Hepatic mRNA expression of the depicted genes was analyzed by qPCR following 49 days of MCDD feeding (left panel, n=5-6/genotype and timepoint). Number of Sox9-positive cells was analyzed by immunohistochemistry and quantified (right panel; n=4-5/genotype and timepoint). (**E**) Representative Sirius Red stainings following 49 days of MCDD feeding. RNA expression is shown relative to untreated controls. Significance was tested by Mann-Whitney test. p values are indicated if significant.. Scale bar=50 µm.

**Supplementary Fig.6. Characterization of Opn-positive cells in livers of MCDD-fed *c-Jun*^Δli^ mice**

(**A, B**) Representative double-immunofluorescence stainings of Opn (green) and Sox9 (red) (**A**) or αSMA (red) (**B**) of livers from mice with the indicated genotypes following 49 days of MCDD feeding. Fluorescence channels are depicted individually (upper three panels) and as Opn/Sox9 (**A**, lower panel) or Opn/αSMA (**B**, lower panel) overlay without DAPI. (**C**) Representative immunohistochemistry of Sox9 (red) and CK-19 (brown) double-positive cells of livers from mice with the indicated genotypes following 49 days of MCDD feeding. Scale bar (immunofluorescence)=25 µm. Scale bar (immunohistochemistry)=10 µm.

**Supplementary Fig.7. Characterization of CD44-positive cells in livers of MCDD-fed *c-Jun*^Δli^ mice**

(**A, B**) Representative double-immunofluorescence stainings of CD44 (green) and Sox9 (red) (**A**) or αSMA (red) (**B**) of livers from mice with the indicated genotypes following 49 days of MCDD feeding. Fluorescence channels are depicted individually (upper three panels) and as CD44/Sox9 (**A**, lower panel) or CD44/αSMA (**B**, lower panel) overlay without DAPI. Scale bar=25 µm.

**Supplementary Fig.8. Phenotype of MCDD-fed mice with broader *c-Jun* deletion (*c-Jun*^Δli*^)**

(**A**) Representative H&E stainings of livers from mice with the indicated genotypes following 0 and 49 days of MCDD feeding. (**B**) Numbers of Ly6G-, CD3-, F4/80- and NKp46-positive cells were analyzed by immunohistochemistry (n=4-13/genotype and timepoint). (**C**) Quantification of Ki67-positive NPLCs by immunohistochemistry (n=4-12/genotype and timepoint). Significance was tested by Mann-Whitney test. p values are indicated if significant.. Scale bar=50 µm.

**Supplementary Fig.9. Characterization of Opn-positive cells in livers of MCDD-fed *c-Jun*^Δli*^ mice**

(**A, B**) Representative double-immunofluorescence stainings of Opn (green) and Sox9 (red) (**A**) or αSMA (red) (**B**) of livers from mice with the indicated genotypes following 49 days of MCDD feeding. Additionally, fluorescence channels are depicted individually (upper three panels) and as Opn/Sox9 (**A**, lower panel) or Opn/αSMA (**B**, lower panel) overlay without DAPI. Co-expression is indicated by red arrows. Scale bar=25 µm.

**Supplementary Fig.10. Expression analysis of Sox9, CD44 and c-Jun- in livers of MCDD-fed *c-Jun*^Δli*^ mice**

(**A, B**) Representative immunohistochemistry of c-Jun (**A**=brown, **B**=red) and Sox9 (**A**=brown) or CK-19 (**B**=brown) double-positive cells of livers from mice with the indicated genotypes following 49 days of MCDD feeding. (**C**) Representative immunohistochemistry of c-Jun (brown) and CD44 (red) double-positive cells of livers from mice with the indicated genotypes following 49 days of MCDD feeding. Co-expression is indicated by red arrows. Scale bar=10 µm.

**Supplementary Fig.11. Characterization of CD44-positive cells in livers of MCDD-fed *c-Jun*^Δli*^ mice**

(**A, B**) Representative double-immunofluorescence stainings of CD44 (green) and Sox9 (red) (**A**) or αSMA (red) (**B**) of livers from mice with the indicated genotypes following 49 days of MCDD feeding. Additionally, fluorescence channels are depicted individually (upper panels) and as CD44/Sox9 (**A**, lower panel) or CD44/αSMA (**B**, lower panel) overlay without DAPI. Co-expression is indicated by red arrows. Scale bar=25 µm.

**Supplementary Fig.12. MCDD-related NASH in *Opn*^-/-^ mice mimics the phenotype of *c-Jun*^Δli*^ mice**

(A) Representative H&E stainings of livers from MCDD-fed mice with the indicated genotypes. (B) NKp46-positive cells were quantified by immunohistochemical staining of liver sections (n=4-8/genotype and time point). Significance was tested by Mann-Whitney test. p values are indicated if significant.. Scale bar=50 µm.
