# Supplementary material for: The transcription factor c-Jun/AP-1 promotes liver fibrosis during non-alcoholic steatohepatitis by regulating Osteopontin expression
Source: Cell Death Differ. 2019 Feb 18;26(9):1688–99. doi: 10.1038/s41418-018-0239-8 (PMC6748141; doi:10.1038/s41418-018-0239-8)
Supplement: Supplementary file 2 — Supplementary Tables [file 41418_2018_239_MOESM2_ESM.docx]

**Supplementary Material**

**Suppl. Table 1**

Antibodies used for immunohistochemistry, –fluorescence and Western Blot

| **Antibody** | **Company** | **Catalogue #** |
| --- | --- | --- |
| Anti-c-Jun | Cell Signaling, Frankfurt, Germany | 9165 |
| Anti- cleaved Casp 3 | Cell Signaling, Frankfurt, Germany | 9661 |
| Anti-Opn | R&D Systems, Abingdon, Great Britain | AF808 |
| Anti-Sox9 | Milipore, Schwalbach, Germany | AB5535 |
| Anti-CD3 | Thermo Fisher Scientific, Rockford, IL, USA | RM-9107 |
| Anti-Ly6G | BD, Heidelberg, Germany | 551459 |
| Anti-F4/80 | Bio-Rad, Munich, Germany | MCA497GA |
| Anti-NKp46 | R&D Systems, Abingdon, Great Britain | AF2225 |
| Anti-CD44 | BD, Heidelberg, Germany | 553131 |
| Anti-Hspa5 | Cell Signaling, Frankfurt, Germany | 3177 |
| Anti-αSMA | DAKO, Jena, Germany | M0851 |
| Anti-Ki67 | Thermo Fisher Scientific, Rockford, IL, USA | MA5-14520 |
| Anti-CK-19 | Kindly provided by R. Kemler |  |
| Anti- β-Actin | Cell Signaling, Frankfurt, Germany | 4967 |
| HRP-conjugated anti-rabbit | Cell Signaling, Frankfurt, Germany | 7074 |
| HRP-conjugated anti-rat | Cell Signaling, Frankfurt, Germany | 7077 |
| HRP-conjugated anti-goat | Zytomed Systems, Berlin, Germany | GHP516G |
| AP-conjugated anti-rabbit | Cell Signaling, Frankfurt, Germany | 7054 |
| AP-conjugated anti-rat | Jackson ImmunoResearch, Cambridge, Great Britain | 112-055-003 |
| Alexa-Fluor 546-conjugated anti-rabbit | Thermo Fisher Scientific, Rockford, IL, USA | A11071 |
| Alexa-Fluor 488-conjugated anti-goat | Thermo Fisher Scientific, Rockford, IL, USA | A11055 |
| Alexa-Fluor 546-conjugated anti-mouse | Thermo Fisher Scientific, Rockford, IL, USA | A11018 |

**Suppl. Table 2**

Primer sequences used for qPCR

| **Gene** | **5’ primer** | **3’ primer** |
| --- | --- | --- |
| *Actin* | AGCCATGTACGTAGCCATCC | CTCTCAGCTGTGGTGGTGAA |
| *18 S* | CGCGGTTCTATTTTGTTGGT | AGTCGGCATCGTTTATGGTC |
| *c Jun* | AAAACCTTGAAAGCGCAAAA | CGCAACCAGTCAAGTTCTCA |
| *Opn* | TGCACCCAGATCCTATAGCC | CTCCATCGTCATCATCATCG |
| *Cd44* | TGGATCCGAATTAGCTGGAC | AGCTTTTTCTTCTGCCCACA |
| *Col1a1* | CTGACTGGAAGAGCGGAGAG | GGGAATCCATCGGTCATGCT |
| *Col3a1* | TGACTGTCCCACGTAAGCAC | GAGGGCCATAGCTGAACTGA |
| *Acta2* | GACTACTGCCGAGCGTGA | GCTGTTATAGGTGGTTTCGTGG |
| *Timp1* | CGAGACCACCTTATACCAGCG | ATGACTGGGGTGTAGGCGTA |
| *Timp2* | TATCTACACGGCCCCCTCTT | ATGGGACAGCGAGTGATCTTG |
| *Des* | GTGCATGAAGAGGAGATCCGT | ATGTTCTTAGCCGCGATGGT |
| *Vim* | AGACCAGAGATGGACAGGTGA | TTGCGCTCCTGAAAAACTGC |
| *Tgfb1* | TGGAGCTGGTGAAACGGAAG | TAGATGGCGTTGTTGCGGT |
| *c Fos* | CCAGTCCTCACCTCTTCCAG | TCCAGCACCAGGTTAATTCC |
| *Fra 1* | AGAGCTGCAGAAGCAGAAGG | CAAGTACGGGTCCTGGAGAA |
| *Fra 2* | CCTCCTGTCTGCCTTGGTTA | AACAGGGTGGTTTTCTTCCC |
| *Gadd153* | GCATGAAGGAGAAGGAGCAG | CTTCCGGAGAGACAGACAGG |
| *Pparγ* | TTTTCAAGGGTGCCAGTTTC | AATCCTTGGCCCTCTGAGAT |
| *Cd36* | GCCAAGCTATTGCGACATGA | AAAAGAATCTCAATGTCCGAGACTT |
| *Dgat2* | TCTTGGGTTATCTCGCTGCT | AACGCCTCATAAAGGCACAC |
| *Cyp2e1* | AGGCTGTCAAGGAGGTGCTA | GGAAGTGTGCCTCTCTTTGG |
| *Nrlh1* | ATGAGGGAGGAGTGTGTGCT | GAAGGAGCGCCTGTTACACT |
| *Fxr* | TTGCAGGGAGAAAACGGAACT | TGAAAATCTCCGCCGAACGA |
| *Cyp7a1* | TGCGAAGGCATTTGGACACA | TGAAAATCTCCGCCGAACGA |
| *Cyp27a1* | AGTGATGAGACAGGAGGGCA | TCCTTGTGCGATGAAGATCCC |
