# Supplementary figures and images for: The transcription factor c-Jun/AP-1 promotes liver fibrosis during non-alcoholic steatohepatitis by regulating Osteopontin expression
Source: Cell Death Differ. 2019 Feb 18;26(9):1688–99. doi: 10.1038/s41418-018-0239-8 (PMC6748141; doi:10.1038/s41418-018-0239-8)

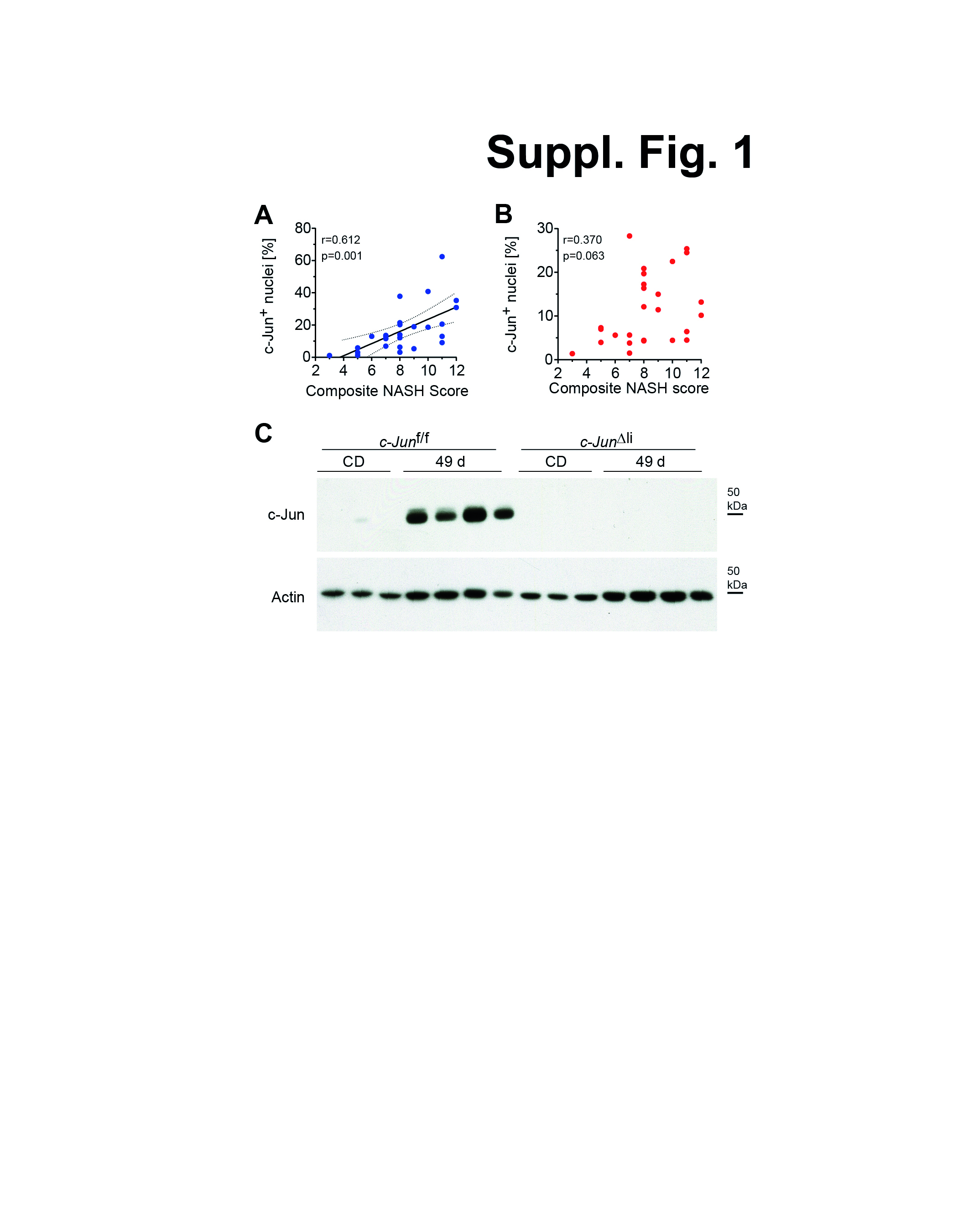

Supplement: Supplementary file 3 — suppl. Fig.1 [file 41418_2018_239_MOESM3_ESM.jpg]

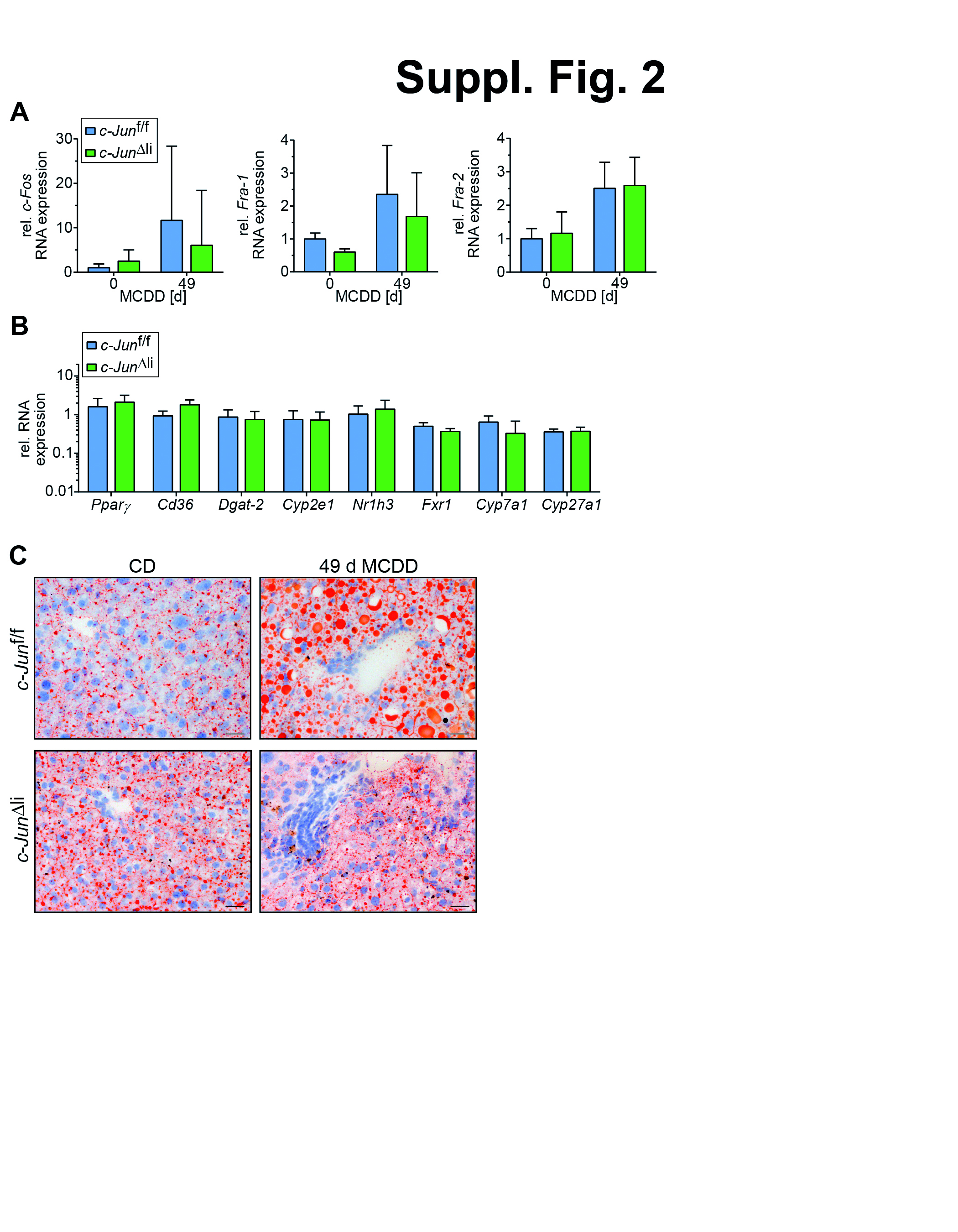

Supplement: Supplementary file 4 — suppl. Fig.2 [file 41418_2018_239_MOESM4_ESM.jpg]

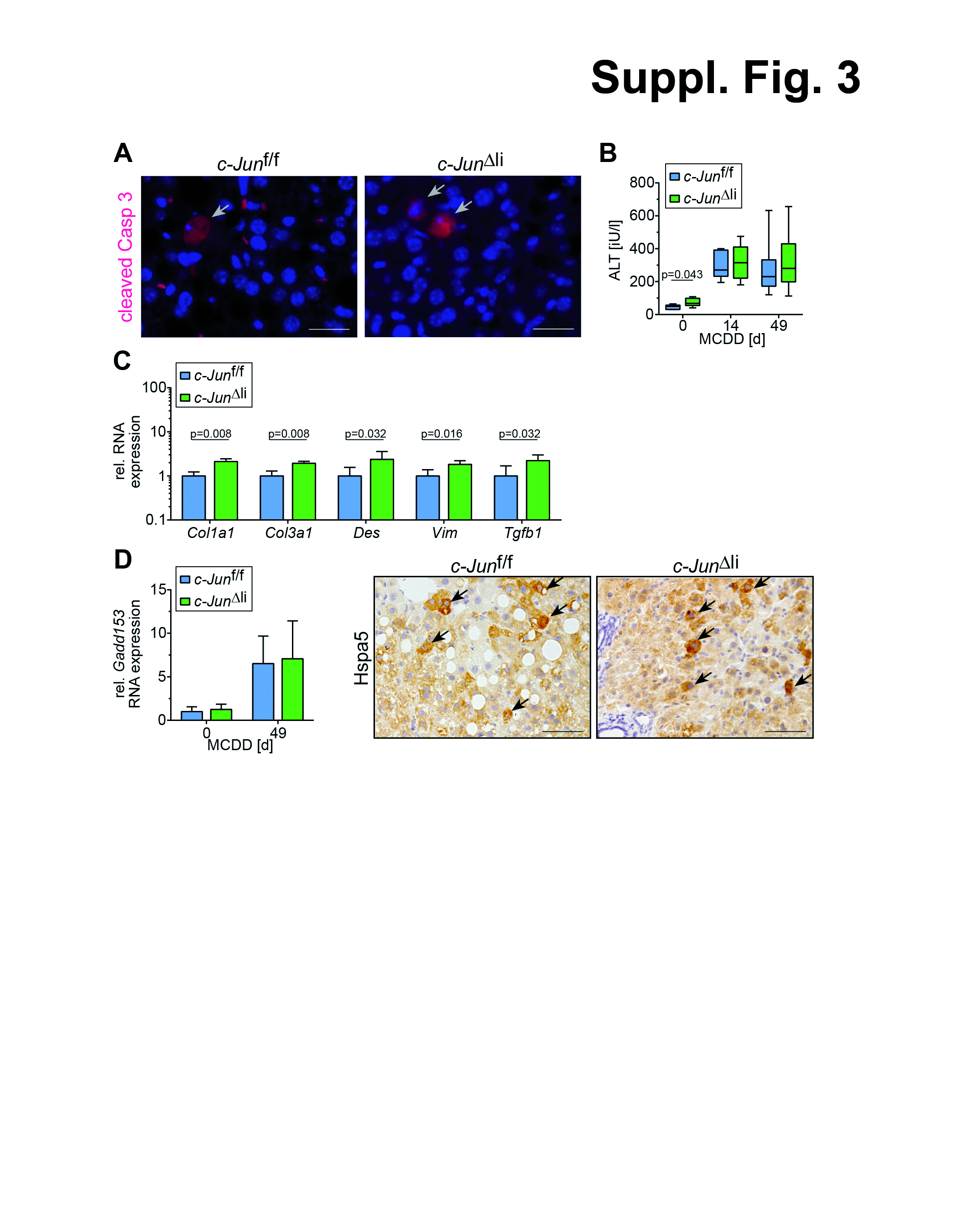

Supplement: Supplementary file 5 — suppl. Fig.3 [file 41418_2018_239_MOESM5_ESM.jpg]

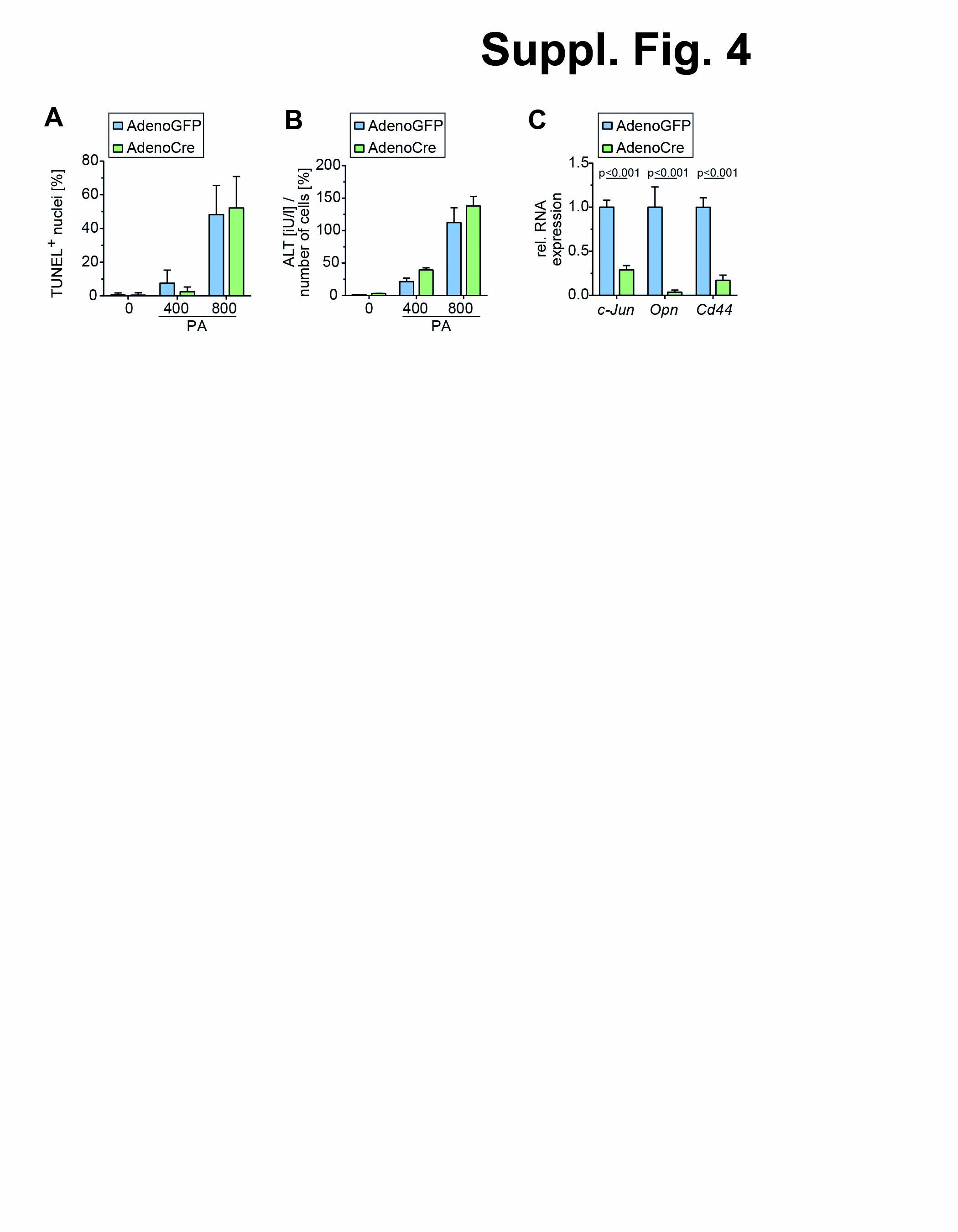

Supplement: Supplementary file 6 — suppl. Fig.4 [file 41418_2018_239_MOESM6_ESM.jpg]

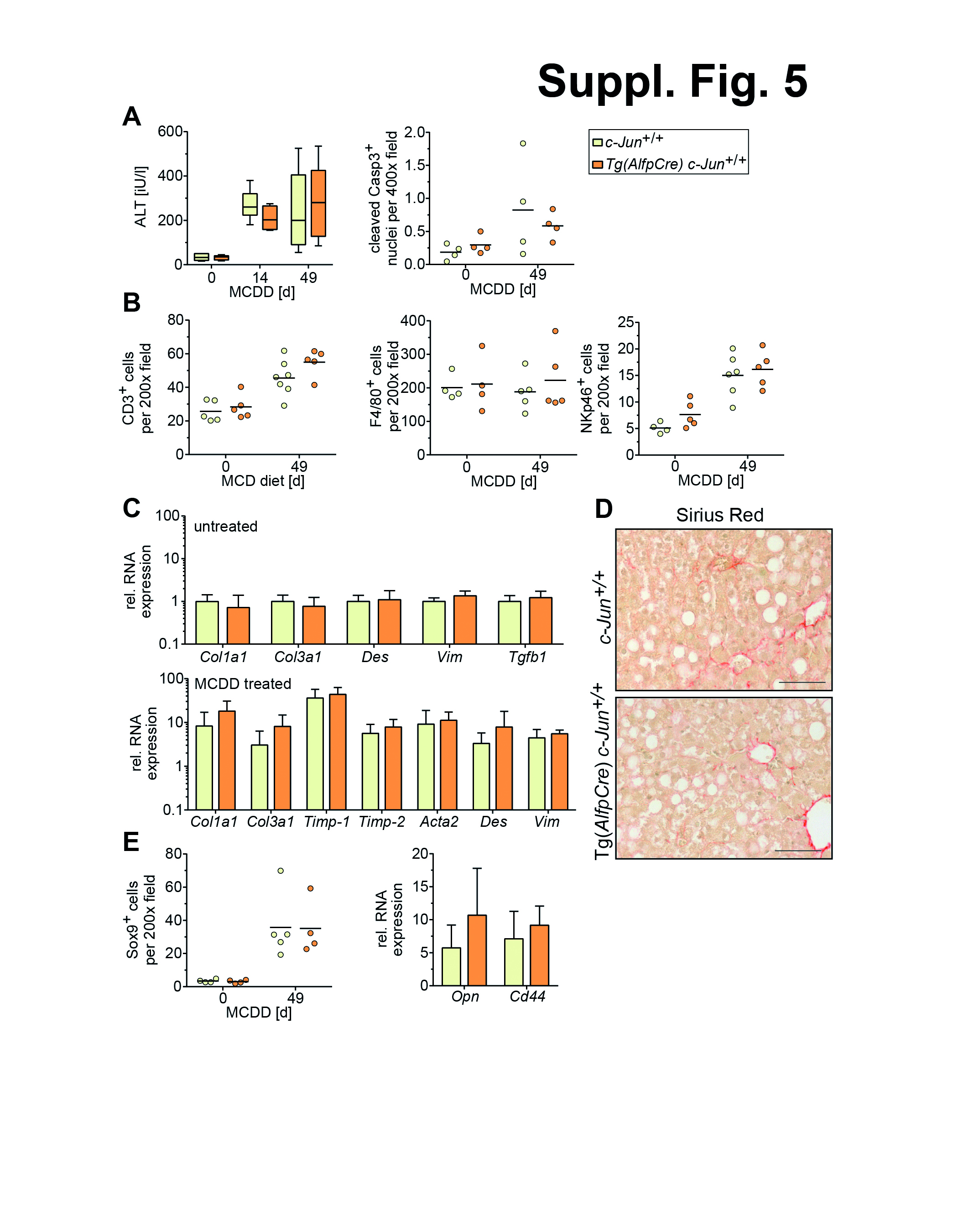

Supplement: Supplementary file 7 — suppl. Fig.5 [file 41418_2018_239_MOESM7_ESM.jpg]

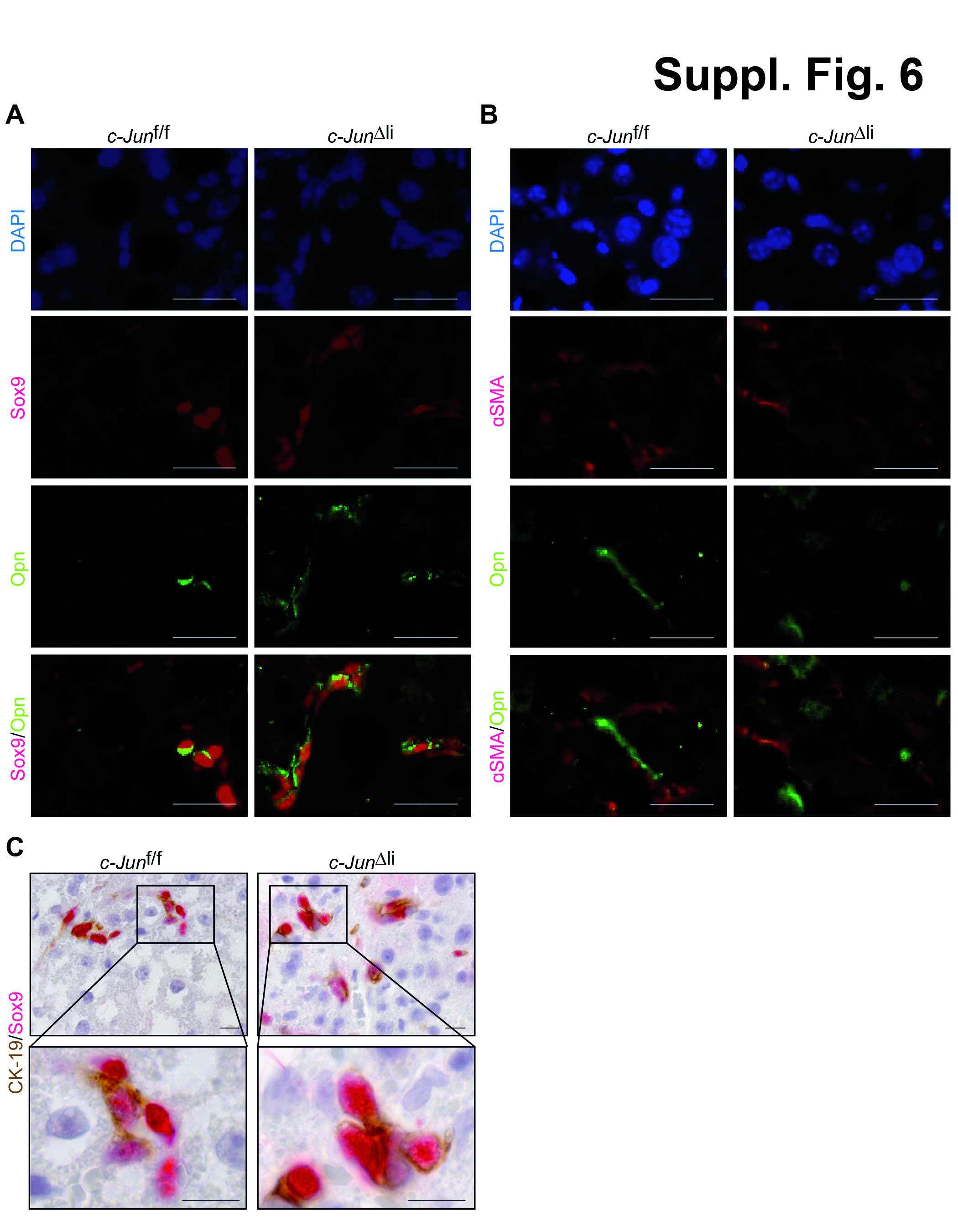

Supplement: Supplementary file 8 — suppl. Fig.6 [file 41418_2018_239_MOESM8_ESM.jpg]

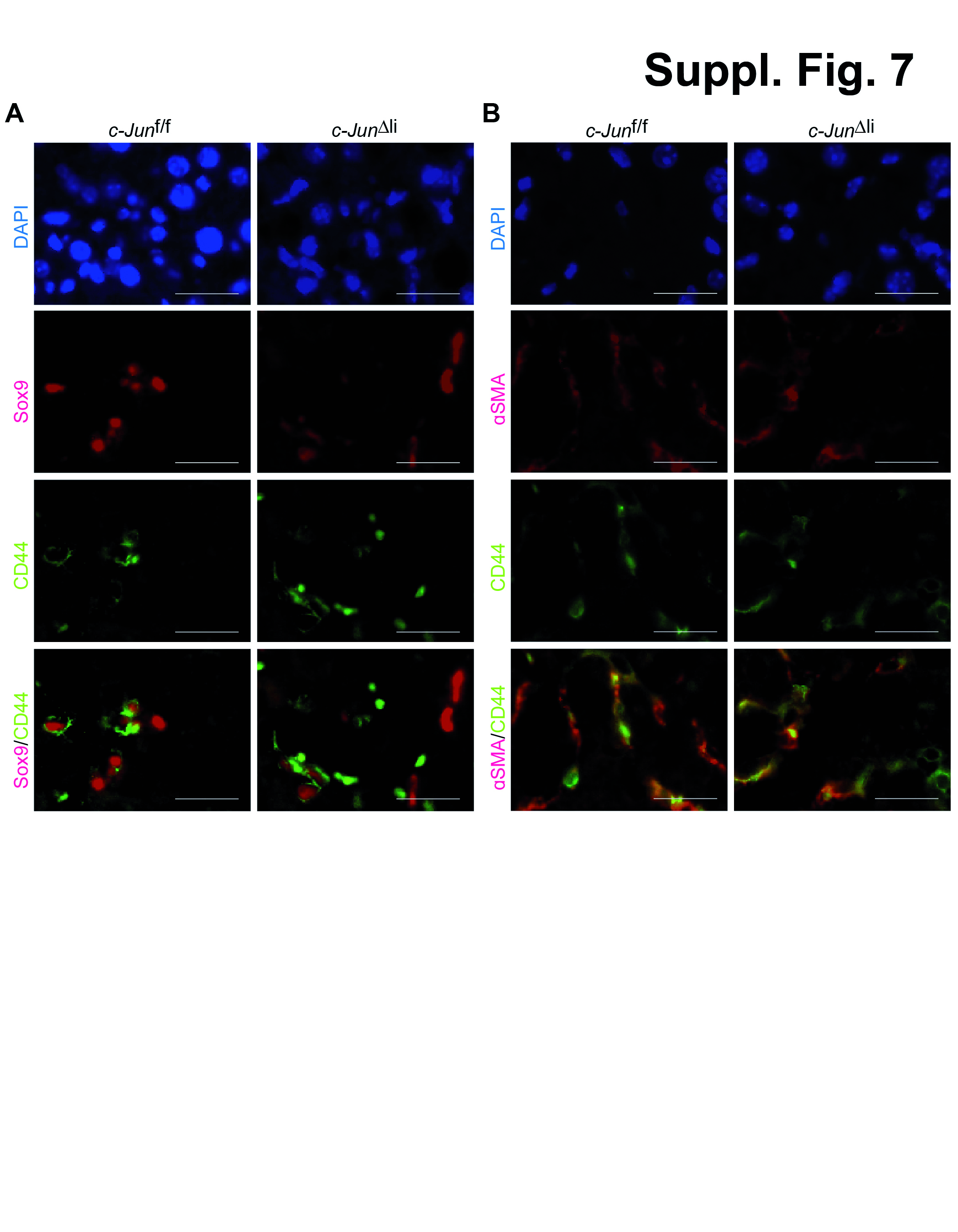

Supplement: Supplementary file 9 — suppl. Fig.7 [file 41418_2018_239_MOESM9_ESM.jpg]

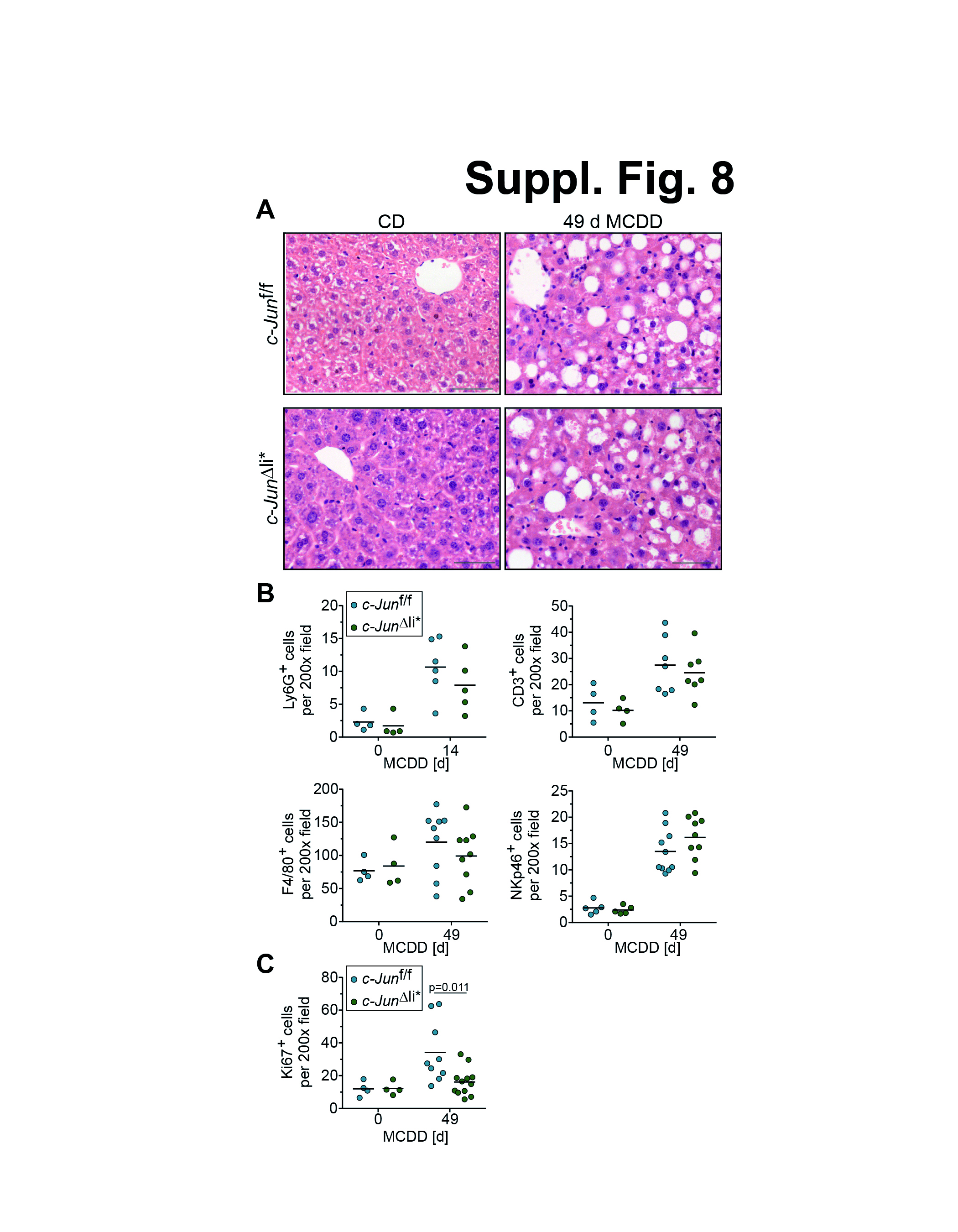

Supplement: Supplementary file 10 — suppl. Fig.8 [file 41418_2018_239_MOESM10_ESM.jpg]

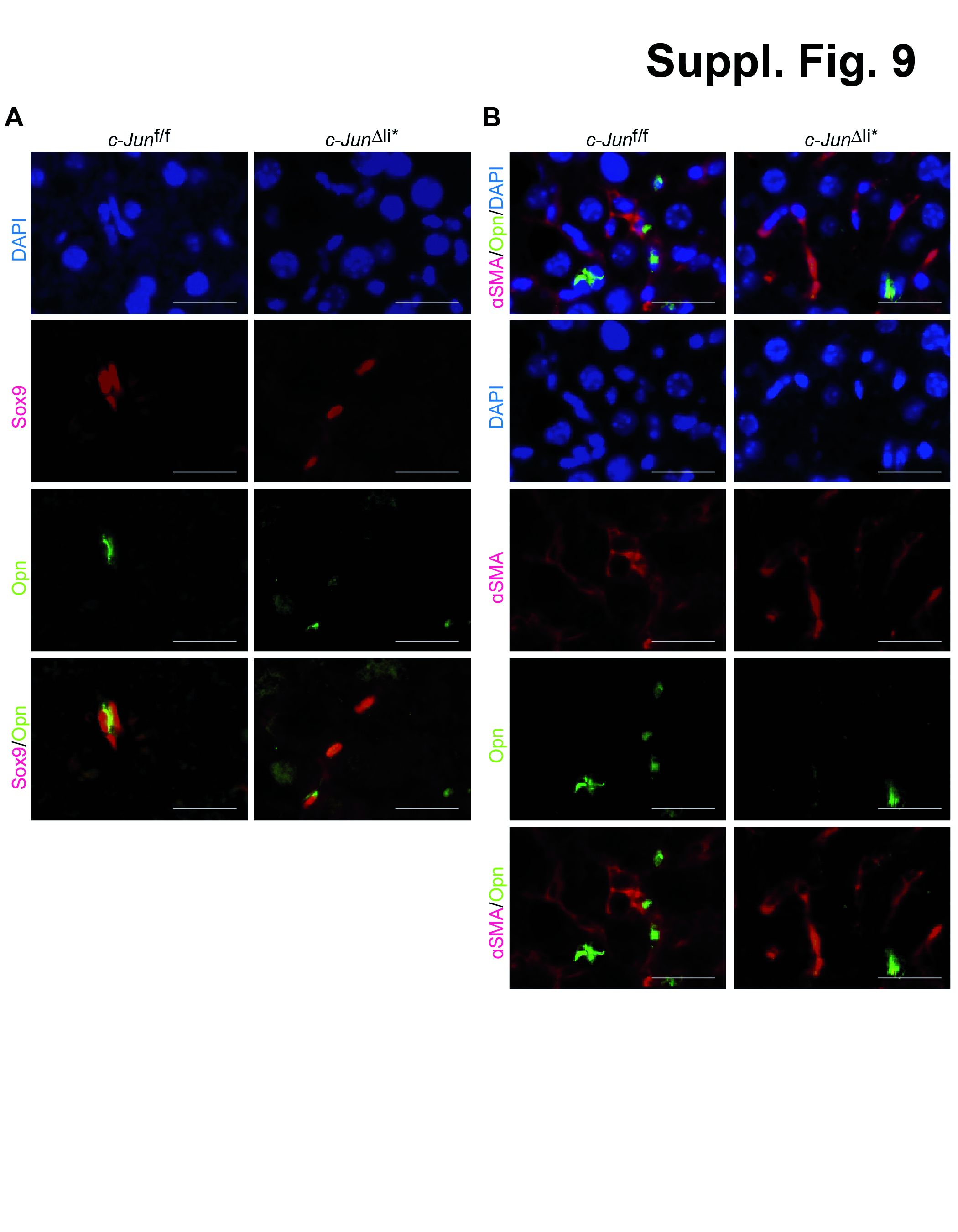

Supplement: Supplementary file 11 — suppl. Fig.9 [file 41418_2018_239_MOESM11_ESM.jpg]

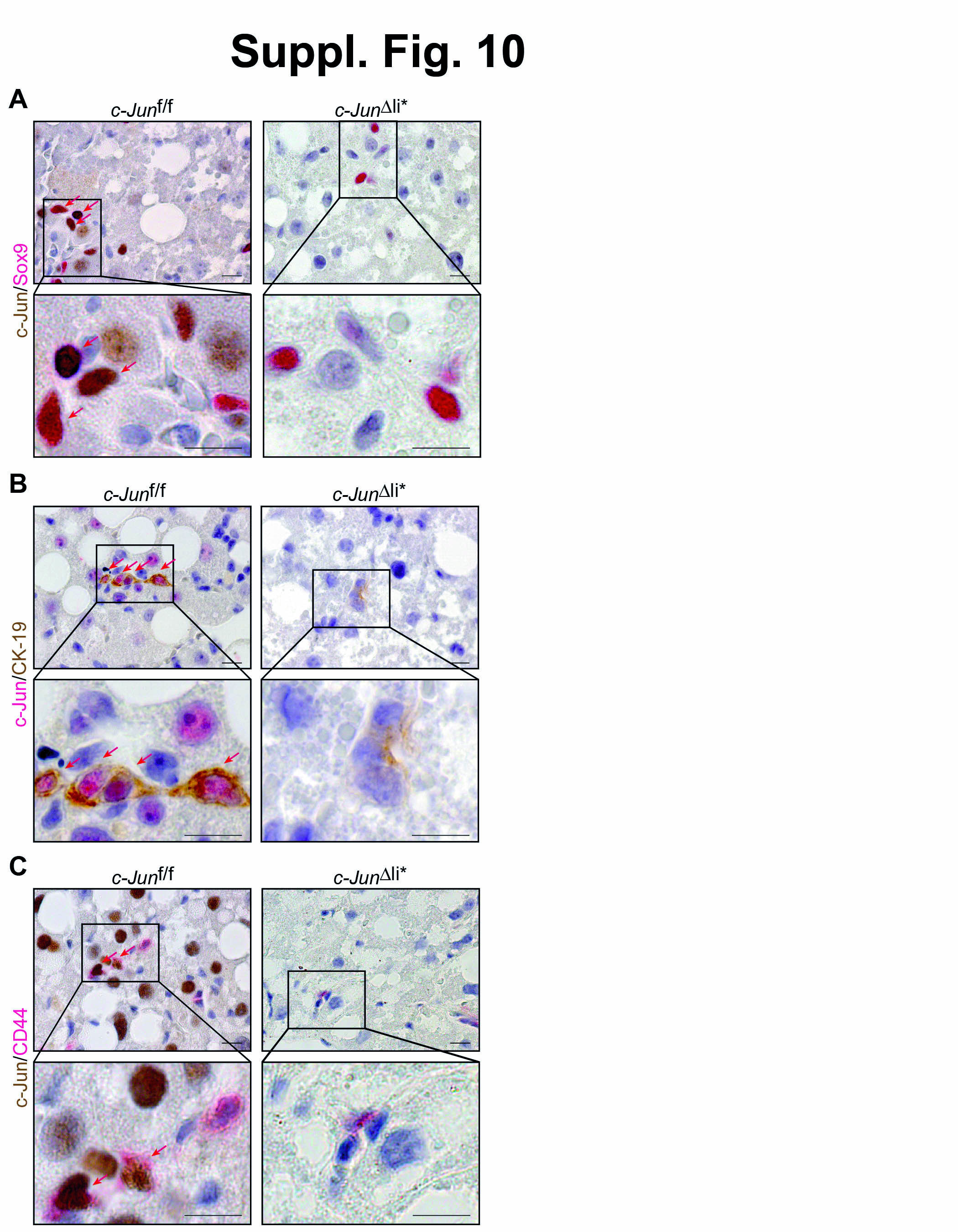

Supplement: Supplementary file 12 — suppl. Fig.10 [file 41418_2018_239_MOESM12_ESM.jpg]

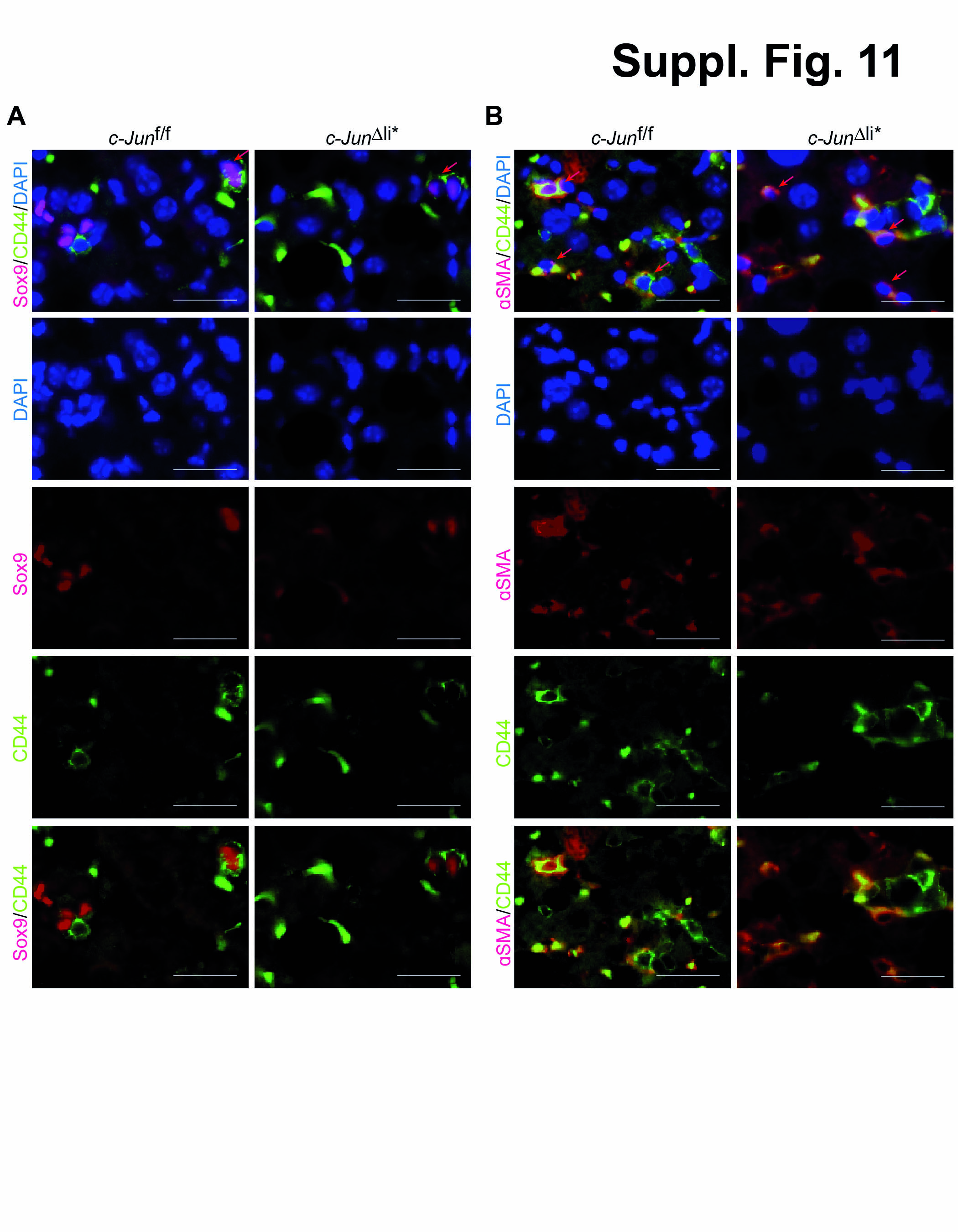

Supplement: Supplementary file 13 — suppl. Fig.11 [file 41418_2018_239_MOESM13_ESM.jpg]

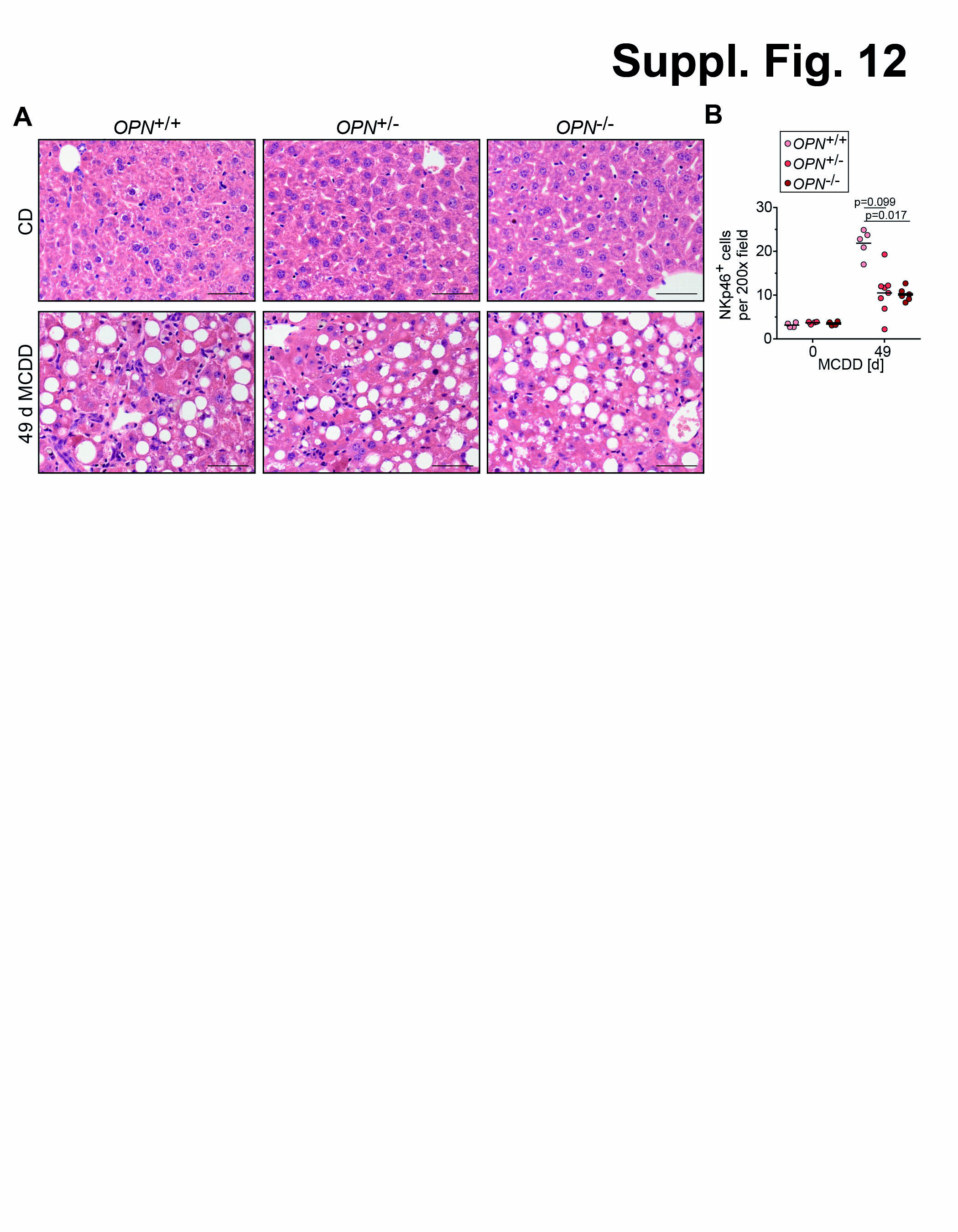

Supplement: Supplementary file 14 — suppl. Fig.12 [file 41418_2018_239_MOESM14_ESM.jpg]
